# Exploring Influencing Factors of Medication Adherence Among Chinese Patients With Alzheimer Disease: Delphi Study Informing Future Artificial Intelligence–Supported Interventions

**DOI:** 10.2196/89508

**Published:** 2026-04-17

**Authors:** Xinyue Zhang, Rafiq Elmansy

**Affiliations:** 1 School of Design and Creative Arts, Loughborough University, Epinal Way, Loughborough, LE11 3TU, United Kingdom, 44 1509 222222; 2 School of Design, University of Leeds, Leeds, England, United Kingdom

**Keywords:** artificial intelligence, medication adherence, adherence, Chinese health care system, online Delphi study, participatory design, design for health, AI-driven medication management

## Abstract

**Background:**

Alzheimer disease (AD) affects cognition, treatment adherence, family connections, and health care resource allocation. Most patients with AD have low adherence to medication therapy due to the limitations associated with cognitive impairment. Therefore, increasing the involvement of patients and their family members in medication management is important to improve treatment outcomes and reduce the burden of care.

**Objective:**

This study explores the potential application of artificial intelligence (AI) in medication management for Chinese patients with early- to mid-stage AD focusing on enhancing medication adherence. The study first predicts and evaluates key factors through an online Delphi study, which provides a basis for their subsequent incorporation into the AI model as input variables to enable prediction of medication-taking behaviors. Since AI research in medication management for this population is still undeveloped, this paper further explores the multiple potentials of AI from a theoretical view, including drug dosage optimization, multidrug interaction detection, and family education support. It will provide a preliminary direction and theoretical basis for the development of an intelligent medication management system in the future.

**Methods:**

The exploratory online Delphi study with no modification predicted the key factors influencing medication adherence. Based on the results, the study confirmed the potential of AI to improve adherence. Participation by 12 experts in 3 rounds systematically assessed the core elements influencing patients’ adherence to their medication.

**Results:**

Family care, social support, environmental factors, emotional support, and patient behaviors were identified as the primary factors influencing medication adherence among Chinese patients with AD. These factors were validated and ranked through iterative Delphi rounds, with family care and social support receiving the highest importance scores. The Wilcoxon signed-rank test indicated no significant difference between rounds (*P*=.06), supporting the stability of the consensus. These findings establish a foundational set of variables for AI systems that predict and enhance medication adherence.

**Conclusions:**

This study highlights the critical factors affecting medication adherence by Chinese patients with AD. It was designed as an exploratory online Delphi study to identify and prioritize key influencing factors, rather than to validate a specific AI-based system, and the findings provide a theoretical foundation for future AI-informed interventions. The results also indicate theoretical potential roles for AI in supporting medication management, such as optimizing drug dosage, detecting multidrug interactions, and enhancing family education.

## Introduction

Progression of the neurodegenerative disorder Alzheimer disease (AD) profoundly impairs cognitive performance and daily activities in older persons, and it has emerged as a critical public health issue due to the aging population worldwide [1,2]. Medication adherence is a critical factor influencing disease outcomes and quality of life in individuals with AD [3]. Nevertheless, deterioration of patients’ cognitive abilities and memory significantly impedes their capacity to precisely and punctually administer medication, leading to poor adherence and suboptimal treatment effects [3-6]. In this context, medication management becomes highly dependent on the sustained involvement of family caregivers. Although family assistance is widely regarded as a key supportive strategy for improving medication adherence, this reliance also substantially increases the burden on family caregivers and does not consistently guarantee sustained and stable medication-taking behaviors [7].

Artificial intelligence (AI) technologies have recently been progressively used in health care, exhibiting significant potential for enhancing drug administration and adherence [8]. The advent of AI offers patients and their families innovative management options that enhance drug adherence and alleviate the stress on carers. Nonetheless, current research has concentrated on the overall patient demographic or the advancement of technology, while investigations into medication adherence to assist with medication management among Chinese patients with AD are scarce [5,6,9,10].

With disease progression and cognitive decline compromising patients’ memory and capacity for independent medication management, family caregivers assume a central role in supporting medication management, underscoring the importance of understanding how caregivers can effectively assist patients in this process. Despite extensive research on medication adherence in AD, existing studies have predominantly focused on patients or formal health care systems, offering limited caregiver-centered insight into how medication management is enacted in everyday practice [11,12]. This is particularly evident in the Chinese context, where caregiving responsibilities are heavily family-based and differ substantially from the assumptions underlying many western health care models [13,14]. Moreover, although AI technologies have shown promise for supporting medication adherence, current research remains largely technology-driven, with insufficient attention to caregivers’ real-world needs and decision-making processes, leaving the practical role of AI in supporting medication management inadequately defined [15]. Accordingly, this study focused on understanding how family caregivers support patients with medication management, with particular attention to how AI-related opportunities may align with caregivers’ everyday knowledge, experiences, and decision-making, as highlighted in existing literature on medication adherence and user-centered health support.

Based on this, this study used an online Delphi study with no modification to assess and validate the key factors affecting medication adherence by Chinese patients with AD and core needs related to patient-centered medication management. In addition, this study aimed to explore the potential opportunities of AI for optimizing medication management and provide a scientific basis for enhancing therapeutic support for patients with AD.

The specific research questions (RQs) are as follows:

- RQ1: What are the influencing factors in medication adherence for Chinese patients with AD?
- RQ2: What is the relative importance of the factors influencing medication adherence by patients with AD?
- RQ3: What adherence factors can inform AI to improve adherence, and how can these factors be effectively used within AI systems?

## Methods

### Delphi Study

This study was designed as an exploratory online Delphi study to identify and prioritize key factors influencing medication use among Chinese patients with AD. The study was not intended to validate an AI-based system but to provide a theoretical foundation for future AI-informed interventions. This study was conducted and reported in accordance with the DelphiSTAR reporting guideline (Checklist 1) [16].

Medication adherence is affected by various intricate elements, including cognitive capacity, familial support, and cultural context [17-21]. This study incorporated professional insights from diverse fields such as gerontology, health, and design. The online Delphi study consensus from panelists incorporates multidisciplinary viewpoints via anonymity rounds, making it particularly suitable for cross-disciplinary research in health studies [22-24].

### Research Framework

This research used a mixed methods approach using the online Delphi study to systematically investigate the primary factors influencing medication adherence by Chinese patients with AD. The study design incorporated qualitative and quantitative in 3 rounds. The rounds were designed to build a consensus of the data. Validity and reliability were confirmed through the data analysis.

The initial round gathered extensive expert opinions through semistructured interviews and developed a preliminary research framework. The data were analyzed using an inductive, bottom-up thematic analysis approach [25], allowing core themes to emerge from the data. This phase was qualitative and focused on identifying key themes. The study used Cohen kappa agreement to verify the credibility and reproducibility of the qualitative data [8]. In this study, interrater reliability was assessed based on 2 randomly selected respondents as an exploratory check of rating consistency. Given the limited sample size, this analysis was not intended to provide a definitive estimate of interrater agreement but rather to support the overall stability of the ratings. The small number of raters represents a limitation and should be considered when interpreting the results [26].

The second round synthesized and validated the outcomes of the first round. The final round amalgamated data from the preceding rounds to ascertain the weights of the factors and achieve consensus. The multiround feedback mechanism and anonymity significantly mitigate bias in group interactions, preserving the independence and impartiality of experts’ viewpoints [27]. Due to the small sample and the fact that the sample did not satisfy a normal distribution, we used Wilcoxon tests [28] to test the differences between the results of round 2 and round 3. The number of Delphi rounds was predefined as three. The process was discontinued after the third round, as the comparison between rounds 2 and 3 showed no statistically significant differences, indicating stability in the expert ratings.

This study developed a theoretical framework for influencing elements in qualitative interviews. At the same time, the quantitative questionnaire empirically validated the real impact of these aspects through accurate measurements and statistical analysis, illustrating the complementary relationship between the two methodologies. Integrating qualitative and quantitative methodologies offers a comprehensive perspective on the subject while enhancing the scientific and practical significance of the findings.

### Panelists

Participants were recruited through online advertisements posted on social media and professional networks from April 2023 to August 2023. The ethics approval and consent to participate subsection describe how informed consent was obtained. We selected 12 participants from an initial pool of 85 candidates who represented a range of professional fields related to AD. All candidates were screened according to predefined inclusion criteria to ensure relevance to the research aims. All 12 experts completed round 1, and the same panel participated in rounds 2 and 3, resulting in a 100% response rate across all rounds.

Specifically, participants were required to meet at least one of the following criteria: (1) having a professional background related to AD or health care practice (eg, clinicans or professionals); (2) possessing substantial practical experience in AD care, such as family or individuals involved in long-term patient support; (3) holding roles related to health policy, organizational decision-making, or service management; and (4) having interdisciplinary experience relevant to health technology, such as design or development of digital or smart health care solutions (including AI). In addition, participants with specific experience applying AI to medication management were given priority. However, due to the emerging nature of the field, no suitable candidates with such expertise were identified.

Notably, although the participants did not have direct practical experience with AI, it was spontaneously raised during discussions with smart packaging experts as a potential support tool. This led to actively and constructively exploring its future applications in health management and packaging interaction.

Table 1 shows the panelist selection.

**Table 1.** Criteria for selection of personnel for the panel of experts.

Filter criteria	Description	Status
Professional health care experience	Participants need to have a professional background related to Alzheimer disease (eg, neurologist).	✓
Practical experience	Candidates with extensive practical experience will be able to provide real-life examples and insights. Examples include patients’ family members.	✓
Policy decision-makers	These include relevant organizational managers and policy decision-makers.	✓
Interdisciplinary experience	This criterion includes health technology (eg, AI^a^ and smart packaging) designers.	✓
AI experience	Experience with practical applications of AI in medicine management is needed.	×
General AI knowledge	Understanding of fundamental concepts, capabilities, and limitations of AI, particularly in relation to health care applications, is needed.	✓

a AI: artificial intelligence.

The final panel consisted of 12 participants, including caregivers (n=7), organizational managers (n=1), neurologists (n=2), and designers or researchers with relevant experience (n=2). This composition ensured diverse perspectives on medication adherence, balancing clinical, practical, managerial, and design insights while remaining feasible in terms of temporal and geographical constraints. All interviews and questionnaires were conducted remotely via the internet, audio-recorded, and transcribed verbatim to ensure data accuracy and study efficiency [29]. The interviews were conducted via Zoom, and surveys were distributed through the Star Questionnaire on WeChat.

The selected panel size is consistent with methodological recommendations for Delphi studies, which generally suggest that expert panels of approximately 10 to 18 individuals can yield stable and meaningful consensus when participants possess relevant domain expertise [30,31]. Accordingly, the inclusion of 12 participants in this study falls within this recommended range and is considered sufficient to support a reliable and scientifically sound consensus process.

### Round 1: Semistructured Interview

This study systematically examined the principal factors influencing medication adherence by patients with AD via semistructured interviews during the qualitative phase. Due to the inherent flexibility, semistructured interviews were suitable for eliciting comprehensive, context-dependent information from respondents regarding family roles, patient behavior patterns, and medication management [32].

The interview questions were developed based on a comprehensive literature review and were informed by theoretical perspectives on medication adherence and user-centered design. Questions assessing participants’ understanding of AD and medications for its treatment (Q1) were included to evaluate caregivers’ baseline health literacy, which is widely recognized as a key determinant of medication adherence in chronic and cognitive conditions [33]. Questions on family involvement (Q2, Q6) were grounded in research indicating that social and emotional support is critical for improving adherence among dementia patients [34,35]. Questions addressing patient behavior and treatment complexity (Q4, Q5) drew upon studies linking cognitive decline and polypharmacy to poor adherence [36,37]. Inquiries into patient experience and smart packaging (Q3, Q7, Q8) were inspired by emerging research on the role of assistive technologies and participatory design in health management [4,17]. A complete list of questions and their corresponding design rationale are provided in Multimedia Appendix 1.

In particular, the interviews included items exploring participants’ experiences and perspectives regarding smart packaging. This focus aimed to understand how packaging design and usability factors might impact medication adherence behaviors among patients with AD and their caregivers.

Core themes were derived from interview data using a coding system and thematic analysis, with the researcher manually extracting codes to ensure depth and accuracy in the analysis. The study validated the interassessor coding consistency using Cohen kappa, an appropriate measure for assessing interassessor agreement while eliminating the influence of random consistency [8,38]. Following validation via this method, the study achieved a high degree of reliability in coding, thereby augmenting the credibility and reproducibility of the data. The detailed evaluation process is provided in Multimedia Appendix 2.

We identified and validated 20 major influences throughout the qualitative study phase, encompassing the primary domains of family roles, patient behavior patterns, and medication management. During the subsequent quantitative research phase, questionnaires were used further to assess the importance and influence of these identified elements.

### Rounds 2 and 3: Questionnaire

**Figure 1.**
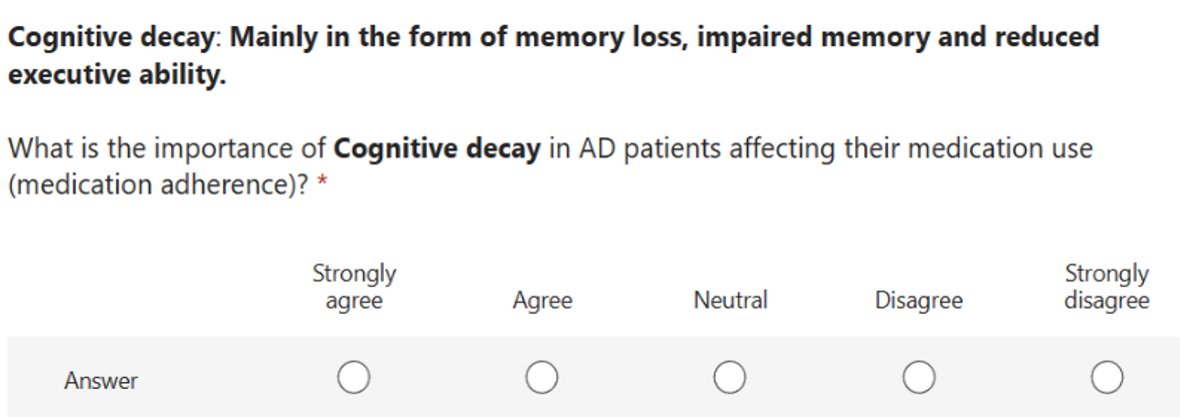
Example of the Likert scale in round 2.

This study evaluated the principal factors influencing medication adherence in patients with AD throughout the quantitative phase using two rounds of assessment using a Likert scale. The Likert scale spans from 1 to 5 and assesses the degree of participant influence on each component [39]. The first round of questionnaires collected participants’ ratings for each factor from which we calculated a mean score. The second round of questionnaires provided feedback on the mean scores from the first round, allowing participants to confirm or adjust their ratings, thereby validating changes and monitoring the dynamics of cognitive processes [36,40]. This methodology integrates the exploratory characteristics of qualitative research with the validation aspects of quantitative research to augment the rigor of the findings. Figures1  2 illustrate examples of the rating format used in Rounds 1 and 2, respectively. In both rounds, the same set of 20 factors corresponding to 20 questions was evaluated. The full list of evaluation factors is provided in Multimedia Appendix 3.

**Figure 2.**
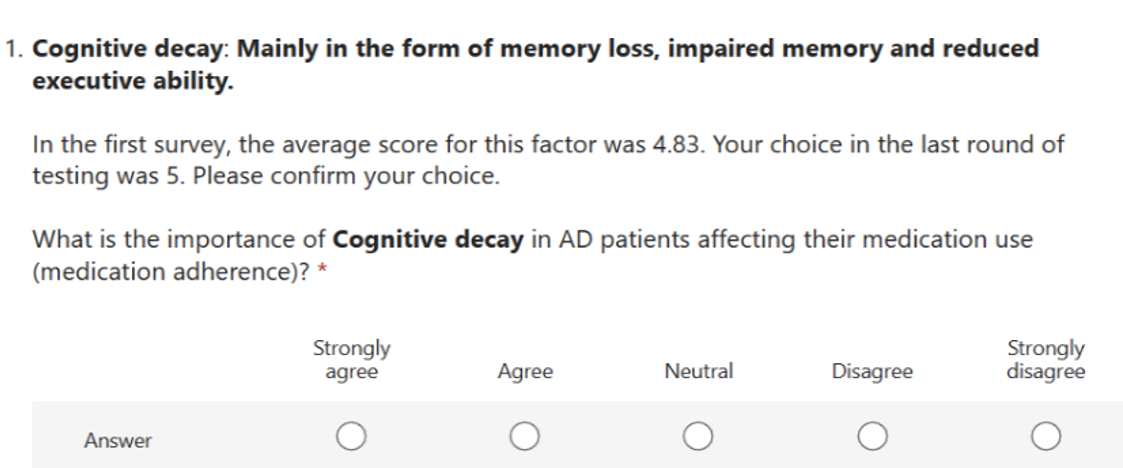
Example of the Likert scale in round 3.

We used the Wilcoxon test to evaluate score changes between the two rounds and to eliminate random variation. This nonparametric statistical technique is appropriate for comparing small samples and data that are not normally distributed [41]. The Wilcoxon test mitigates the influence of outliers on analytical outcomes by ordering the data, enhancing the results’ robustness and reliability [42,43].

### Ethical Considerations

This study was approved by the Ethics Committee of the University of Leeds (approval number: FAHC 22‐002) and was conducted in accordance with the principles of the Declaration of Helsinki. All participants were provided with an information sheet outlining the study aims, procedures, voluntary nature of participation, and data protection measures. Written informed consent was obtained prior to participation through an opt-in procedure. Participants were informed of their right to withdraw at any time without penalty and the publication of their anonymized data.

Because the same individuals took part in both the questionnaire and interviews, the study data were treated as confidential rather than anonymous. The questionnaire collected personal data (name, email, profession, and expertise). Participants’ identities were known to the researcher during data collection. Identifying information was removed from interview transcripts, and pseudonyms were used in all reporting. No compensation was offered. Data were stored securely on password-protected institutional devices in accordance with institutional data protection policies and UK GDPR [44].

## Results

### Round 1: Semistructured Interviews

This study examined the participation of family members of patients with AD in treatment, medication experiences, and patient behavior through remote semistructured interviews with 12 individuals. Each interview lasted 30 minutes to 45 minutes and was audio-recorded for later analysis. A categorical method was used to enhance the analysis of participants’ perspectives and experiences [45]. Due to a small sample restriction, codes were manually derived from the semistructured interview data. Simultaneously, the integration of interviews with the literature analysis yielded 4 principal categories and 20 subcategories about medication adherence: familial and social support, patient self-management, health care service design, and problems in health care management. Table 2 illustrates the categories of the theme analysis, whereas Multimedia Appendix 2 presents the methodology for extracting the interview codes.

**Table 2.** Theme analysis.

Themes and subthemes	Code
Family and social support	
Family and social support	Home care/daily care
Emotional support	Family companionship, treatment attitudes, emotional comprehension (eg, encouragement)
Therapeutic assistance	Family supervision, medication management, doctor-patient communication
Caregiver education	Nursing skills, effective communication, reduction of psychological burden, understanding of the condition
Family environment	Stability, motivation of family to cooperate with treatment, living alone, living situation
Patient self-management	
Cognitive decay	Disease progression, memory impairment, diminished executive ability, memory impairment
Treatment awareness	Resisting medication, lack of awareness
Complexity of medication regimens	Polypharmacy, dosing frequency, units per dose, and non-oral routes
Treatment motivation	Mental health, treatment beliefs and attitudes, treatment engagement
Habits and behavior	Smoking and drinking
Medical management challenge	
Progression of illness	Stage of illness, cognitive degeneration, aggravated, change of treatment program
Clinical progression	Therapeutic strategies: polypharmacy, drug type
Therapeutic effects	Drug side effects, drug effect, delayed drug effects, precise dosage
Doctor-patient communication	Communication clarity, information asymmetry, inconsistent medical advice, limited consultation time, caregiver involvement in decision-making, understanding of medication instructions
Financial factors	Treatment costs, financial burden
Medical service design	
Assistive tools	Application, pill box dividers, smart packaging
Technology and innovation	AI^a^, AR^b^, image processes, teletherapy, real-time monitoring
User demands and experience	Accessible design, information accessibility, messaging and understandability
Human-computer interaction	Drug packaging and labeling design, social support platform, health care accessibility and flexibility, medication management applications, supervise medication, filtering information
Cultural awareness	Weak culture awareness

a AI: artificial intelligence.

b AR: artificial reality.

### Interrater Reliability: Calculation of Cohen Kappa Agreement

After concluding the first round of code analyses and assessments for this study, two questionnaire results (R1 and R2) were chosen randomly to test the consistency of scores. Banerjee et al [8] suggested that random sampling reduces bias and increases the sample’s representativeness, which can increase the findings’ credibility to some degree. Table 3 compares the code categories of participants R1 and R2 that are mapped to each other and correspond to the study’s codes.

**Table 3.** The comparison between the code categories of researcher R1 and R2.

Code categories	R2		
	0	1	Sum (%)
R1			
0	4	2	6 (0.25)
1	3	15	18 (0.75)
Sum (%)	7 (0.3)	17 (0.7)	24

In a binary system, “1” means agreeing with the initial code, and “0” means disagreeing with the initial code. Multimedia Appendix 2 displays the accord between the first and second evaluators regarding the initial codes. The analysis is shown in Table 3.

The observed rater agreement, Pa, is calculated first. This value may involve coincidence:

Pa=(N00+N11)/24

Pa=(4+15)/24=0.95

Multimedia Appendix 2 displays the likelihood that both raters will concur. The first opportunity is when they concur on the researcher’s initial code. This time, they record the same code (or a similar one). The second opportunity arises when respondents concur with one another but disagree with the researcher. The probability of coincidental agreement (Pe) is determined in the second stage. The following equation is used to calculate this probability:

PE=(R1_0_/6)×(R2_0_/7)+(R1_1_/17)×(R2_1_/18)

Pe=0.25×0.3+0.71×0.75=0.6075≈0.61

where Pe is the probability of assuming chance agreement, R1_0_represents the total codes where the first rater disagrees, R1_1_ is the max number of codes the initial rater competes with, R2_0_ is the number of codes the second rater disagrees with, and R2_1_ is the number of codes the second rater accepted.

According to these equations, there is a 60% possibility that two raters will coincide by chance. The final Cohen kappa value was therefore: kappa=(Pa-Pe)/(1-Pe)=(0.95‐0.61)/(1‐0.61)=0.87≈0.9.

Cohen kappa ranges from 0 to 1, with 0 indicating no agreement and 1 indicating complete agreement. Based on this ratio, we found a value of 0.9. Raters agreed 90% of the time. Landis and Kock [46] calculated the kappa value to assess the Cohen value for data reliability. The “Strength of agreement” column in Table 4 shows that 0.9 represents almost flawless reliability. The protocol data analysis is in Table 4.

**Table 4.** Cohen kappa reliability and agreement values [46].

Value for reliability	Strength of agreement
<0.00	Poor
0.00‐0.20	Slight
0.21‐0.40	Fair
0.41‐0.60	Moderate
0.61‐0.80	Substantial
0.81‐1.00	Almost perfect

### Round 2: Questionnaire

The first Likert scale assessed 20 adherence variables for patients with AD. Using the Likert scale, the mean score for each question was calculated and ranked from highest to lowest to ascertain the significance of the factor. After the first round of quantitative evaluation, the mean score for each factor was determined, as shown in Table 5. The key factors were family care and social support, habits and behaviors, emotional support, home environment, treatment assistance, and doctor-patient communication.

**Table 5.** Scale for assessing scores of influencing factors in Chinese patients with Alzheimer disease according to the Likert scale in round 2.

Number	Keywords	Score, mean
1	Family care and social support	4.83
2	Habits and behaviors	4.42
3	Emotional support	4.33
4	Family environment	4.33
5	Therapeutic assistance	4.25
6	Doctor-patient communication	4.17
7	Progression of illness	4.08
8	Cognitive decay	4.08
9	Treatment awareness	4.08
10	Financial factors	4
11	Complexity of medication regimens	4
12	Cultural awareness	3.92
13	Therapeutic effects	3.92
14	Caregiver education	3.75
15	Human-computer interaction	3.67
16	User demands and experience	3.67
17	Treatment motivation	3.58
18	Clinical progression	3.58
19	Assistive tools	3.08
20	Technology and innovation	2.92

### Round 3: Questionnaire

We used the same scale for the third evaluation. It gave users the mean scores to evaluate the significance of each subcategory collected in the initial round. In addition, they could view the previous responses during the second round. The procedure was devised to allow panelists to review their answers from the previous round of testing and change their minds if necessary. By assessing adherence factors in patients with AD twice using a Likert scale, it was possible to assess the stability of relevant adherence influencing factors, to assess the efficacy and significance of the intervention, and to provide support for the individualization of the intervention and quality control of the study [39,47].

### Evaluation of Test Results Using the Wilcoxon Test

We used the Wilcoxon test to compare arithmetic results between the second and third phases to assess consistency and inconsistency, facilitate the foundation of consensus, and exclude random variation, thereby enhancing the credibility of the study results [41,42]. Comparing the results of the two testing sessions revealed that the panelists altered their responses, resulting in changes in the SDs of some factors (Table 6) and demonstrating consensus among the panelists regarding the significance of each factor influencing adherence.

**Table 6.** Standard deviation (SD) results of the second and third round tests.

Influencing factors	Round 2 SD	Round 3 SD
Family care and social support	0.37268	0.64010
Therapeutic assistance	0.55277	0.5
Caregiver education	1.01036	0.75920
Family environment	0.62361	0.89753
Emotional support	1.14261	0.43301
Cognitive decay	0.98601	0.49301
Treatment awareness	0.70711	0.70711
Complexity of medication regimens	0.70711	0.79931
Treatment motivation	1.11803	0.64010
Habits and behaviors	0.64010	0.68718
Progression of illness	0.62361	0.49301
Clinical progression	1.02740	0.79931
Therapeutic effects	1.18732	0.79931
Doctor-patient communication	0.98601	0.72169
Financial factors	1.29099	1.06719
Assistive tools	1.01036	1
Technology and innovation	0.75920	1.23322
User demands and experience	1.03749	0.86201
Human-computer interaction	0.74536	0.64010
Cultural awareness	0.86201	0.59512

Wilcoxon signed-rank tests were performed on the SDs from the second and third rounds to determine chance-excluded changes. The data in Table 5 were tested with SPSS (IBM Corp).

Of the values in Table 7, 14 had a negative ranking, indicating that the SD value in round 3 was less than in round 2. The 5 values that were ranked positively indicate that the SD increased in the second round. One of the values had a tied position, meaning that the second and third phases had the same value. The null hypothesis for this study’s Wilcoxon test was "There is no change in standard deviation between the first and second rounds, and the difference between the first and second rounds follows a symmetric distribution around zero.” The Wilcoxon signed-rank test results for *round 3 SD-round 2 SD* were *z*=–1.851 (*P*=.06) based on positive ranks.

**Table 7.** Statistics for the Wilcoxon signed-rank test.

Round 3 SD-round 2 SD	Results (n=20), n	Mean ranking	Sum of rankings
Negative rankings	14^a^	10.07	141.00
Positive rankings	5^b^	9.80	49.00
Ties	1^c^	—^d^	—

a Round 2 SD<round 1 SD.

b Round 2 SD>round 1 SD

c Round 2 SD=round 1 SD.

d Not applicable.

For all statistical analyses, a *P* value <.05 was considered statistically significant [48,49]. The Wilcoxon signed-rank test yielded a *P* value of .06, which exceeds the predefined significance threshold. The null hypothesis of no significant difference in SDs in rounds 2 and 3 cannot be rejected, indicating stability in the distribution of expert ratings across rounds. Therefore, the results remained consistent with those obtained in round 2 (more details in Table 5).

## Discussion

### Individual Perspectives: Medication Complexity and Individualized Needs

Pharmaceutical treatment of AD is notably complex, particularly with polypharmacy, where adherence poses a considerable challenge. Polypharmacy is a significant problem affecting drug adherence by patients with AD, as evidenced by the intricate nature of treatment regimens. Patients frequently need to administer numerous drugs, which imposes significant demands on the medication management capabilities of both patients and their families [34,37,50].

The timing and dosage of each medicine is different, and it takes much effort on my part to keep track and monitor, especially the measurement, timing, and temperature of the herbs.

[C5]

Moreover, drug-drug interactions exacerbate therapy uncertainty, especially for patients with AD who have various comorbidities [35]. Patients with comorbidities must manage both AD drugs and concurrent prescriptions for other diseases, such as antihypertensives or antidiabetics. This simultaneous treatment may result in pharmacokinetic and pharmacodynamic interactions that could provoke adverse reactions and heighten therapeutic risks [51].

My mother has diabetes and high blood pressure, and she needs so many different types of medication that I am concerned that they will conflict.

[C6]

Personal variations also substantially influence medication treatment. Hampel et al [52] observed that patients with mild and severe AD need distinct drug combinations owing to variations in phenotypic and underlying pathology. Nonetheless, the drug dose is frequently assessed in healthy older individuals during current drug development, a bias that overlooks patients’ specific requirements [53]. Consequently, improving drug doses and treatment protocols to mitigate potential drug interactions and enhance the precision and customization of therapy has emerged as a research priority.

### Family Perspectives: Caregiver Burden and Emotional Support

In the Chinese cultural framework, families significantly influence the care of patients with AD. Due to the traditional culture of “filial piety,” family members frequently act as the principal carers for patients [54]. As the disease advances, the patient’s self-care capacity diminishes [55], requiring more support from family carers, particularly for medication administration [56,57].

Our family lives in a rural area, and it’s hard to follow the doctor’s advice, especially since medication usage is often confusing.

[C2]

The family’s emotional condition significantly influences the patient’s motivation and adherence to medicine [58]. Emotional support alleviates patients’ psychological distress and enhances their acceptance of and compliance with treatment [59]. Long-term care obligations impose significant psychological and physical strain on families, diminishing the efficacy of family oversight and potentially resulting in neglect of drug management. Furthermore, research indicates that the negative feelings of family members can elicit suspicion or resistance in patients, thereby impacting treatment outcomes [60].

Caring for patients requires so much time commitment that I have felt exhausted, and several times I have forgotten to remind my father to take his medication, which makes me crazy.

[C5]

### Societal Perspective: Inadequate Resources and Cultural Influences

Elevated levels of social support have been demonstrated to markedly enhance patients’ adherence to medicine [61]. The disproportionate allocation of health care resources and inadequate public awareness of AD in China’s eastern and western regions substantially impact patient diagnosis and treatment rates [9,62].

Our family lives in a rural area, and it is hard to follow the doctor’s advice, especially since medication usage is often confusing.

[C2]

Cultural influences are also major elements that affect drug adherence. Certain family members of patients reject western medication in favor of herbal remedies, citing worries regarding the adverse effects associated with western pharmaceuticals [63]. Moreover, families’ inadequate comprehension of medical information and insufficient recognition of treatment significance undermine drug adherence [64]. This indicates that enhancing family members’ awareness and acceptance of medication management through education and information is essential for addressing the issue [65].

### Technological Perspective: Emerging Smart Technologies and the Potential of AI

Recent technological advancements have introduced new opportunities for enhancing medication adherence among patients with AD. Smart packaging, wearable monitoring devices, and mobile health apps have demonstrated potential for tracking medication-taking behaviors, sending reminders, and providing dynamic feedback to users [4,17]. These innovations allow for real-time behavioral data collection, offering the possibility of more timely and personalized adherence interventions.

I think if the medicine box could remind us automatically, it would be much easier. Sometimes we forget, and by the time we remember, it’s already too late.

[C5]

Building upon these technological foundations, AI offers promising pathways to enhance medication management further [5,6]. By analyzing real-time adherence data captured through smart technologies, AI-driven systems could predict adherence risks, deliver customized reminders, optimize drug regimens (precision therapy), and provide tailored educational support to caregivers and patients [35,52,66,67]. This integration could significantly strengthen home-based care practices for AD management.

### Opportunities and Challenges for AI

**Figure 3.**
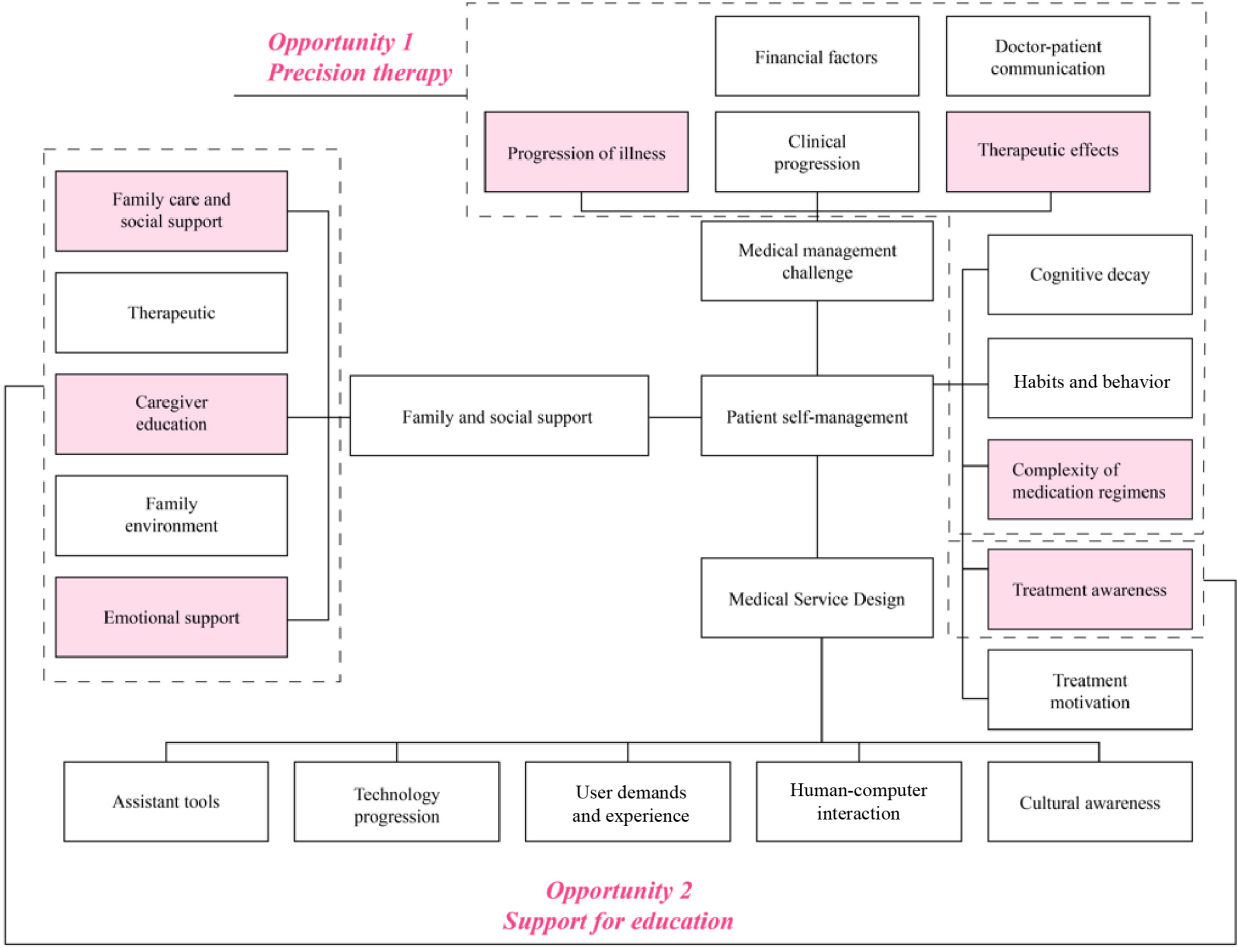
Opportunities for artificial intelligence (AI) drug dose prediction using deep learning and machine learning to assist family members with medication management.

To address how the identified adherence-related factors may inform future AI-supported interventions, Figure 3 presents a conceptual map. Based on the identified adherence-related factors, two overarching opportunities for AI-supported intervention were identified: precision therapy and AI-enabled educational support. This framework does not represent an implemented technical system but rather illustrates how factors identified through the Delphi study could theoretically be operationalized within AI-assisted medication management.

First, factors such as medication complexity, disease progression, and treatment variability highlight the potential role of precision therapy in supporting personalized medication management. These findings suggest that AI systems could, in principle, assist with dosage optimization and risk identification based on patient-specific characteristics [35,52,53,66,67].

Second, factors related to family support, treatment awareness, caregiver education, and emotional burden point to the potential value of AI-based educational and supportive functions. Such systems may support caregivers through tailored information delivery and decision support, thereby addressing gaps in knowledge and reducing caregiving burden [68,69].

It should be emphasized that these opportunities are conceptual rather than technical implementations. Practical challenges related to data availability and ethical considerations currently limit direct operationalization [70,71]. The proposed framework therefore serves as a theoretical foundation to inform future system design and empirical research.

Participants’ insights into smart packaging revealed that such technologies could not only enhance medication adherence through design interventions but also serve as critical data collection touchpoints. Real-time tracking of medication behaviors through smart packaging can generate valuable adherence data, providing a foundation for developing future AI-driven systems.

Furthermore, participants’ reflections highlighted that the requirements and barriers existing at the individual, family, and societal levels significantly influence medication management practices. These multilayered needs inform the design of AI applications, suggesting that future AI-driven interventions should focus on precision therapy and intelligent caregiving and incorporate educational support better to address the complex dynamics of AD care.

### Precision Therapy

Deep learning (DL)–based drug dose prediction can introduce precision medicine to patient-centered drug management [72]. Precision medicine is the process of tailoring medicine to the health status of each patient in order to maximize the quality of health care [73,74].

The personalized treatment of AD via precision medicine has tremendous potential. First, precision medicine can more effectively address the challenges of polydrug management posed by the simultaneous administration of multiple medications [66,67,75]. In their study, Keine et al [35] hypothesized that polypharmacy would increase the risk of adverse drug reactions induced by drug interactions. The polypharmacy faced by older adults with AD is reflected in the multidrug treatment of AD and the need to take multiple medications to maintain life due to various underlying diseases. This emphasizes the imperative need for personalized drug management (ie, more precise drug dosage and drug action detection) to develop a more personalized and accurate drug management strategy. Second, individual differences are biased [52]. The concept of *precision medicine* proposed by Yiannopoulou and Papageorgiou [76] seeks to target the unique drug requirements of each patient to minimize the presence of risk factors and optimize the treatment of comorbidities. When developing drugs for AD, the dosage of drugs is frequently measured against the standard of healthy older adults. In his study, Xing [53] noted that the gender disparity among patients with AD will become an essential factor influencing precise and individualized treatment.

Precision treatment measurement management is inseparable from AI technologies for detection and prediction, such as machine learning (ML) and DL. In the field of patient medication management, AI presents numerous opportunities for the implementation of precision medicine. AI uses methods based on ML and DL to conduct simulation experiments with different concentrations to evaluate the effects of drugs on patients and detect differences in individual patient conditions [77,78]. Alternatively, ML classification methods can be used to screen and classify various drug collections and predict deleterious effects [79]. However, ethical considerations must be considered when using genetic or biomarker testing for early risk assessment and detection [80].

Precision medicine provides an important technological foundation and developmental direction for supporting family members when assisting with medication management. However, its current applications remain largely confined to clinical and research settings, with insufficient consideration of the roles and decision-making contexts of caregivers in everyday medication management.

### Support for Education

AI-based educational support for AD can be helpful in several ways. Munteanu et al [68] pointed out that modern AI technology based on computer vision can provide personalized assistance to patients with AD. For example, Sharma and Rani [69] proposed a health internet framework based on DL to provide decision-making suggestions for patients with AD and their families. The framework integrates anomaly-tracking methods and Internet of Things–based assistance mechanisms to support patients with making correct decisions by informing them of incorrect decisions and providing reinforcement and supportive assistance.

AI-based educational support demonstrates clear potential to assist family caregivers by providing accessible information and decision-related guidance in the context of AD care. However, it is essential to note that, although AI plays a vital role in AD education and support, AI tools as supplementary tools cannot replace the role of doctors and professional medical teams [70,71]. This involves medical ethics responsibility.

### Challenges

Although the development of AI in the medical field has excellent prospects, it still faces some challenges. First, the reliability of AI-based systems remains a key concern, particularly in medication management, where inaccurate predictions or system errors may lead to inappropriate dosing or missed medication [81,82].

Second, the effective use of AI technologies requires a level of digital literacy and sustained engagement that may not be readily achievable among family caregivers of people with AD in China. Many caregivers have limited experience with digital health technologies, which may reduce system adoption, increase cognitive burden, and compromise long-term usability [83].

In addition, the application of AI in medication management raises broader ethical and social concerns. These include questions surrounding responsibility and accountability when AI-generated recommendations are followed, as well as the risk of over-reliance on automated systems in situations that require human judgment and emotional sensitivity [84,85]. Such challenges highlight that AI-based solutions should function as supportive tools rather than replacements for human caregiving and their implementation must be carefully aligned with real-world caregiving practices and ethical considerations.

### Future Studies

Future research should concentrate on integrating precision medicine principles with AI technologies to address the requirements of personalized drug management for AD patients. Priority must be given to optimizing drug dosage, modifying treatment protocols, and identifying multidrug interactions via AI while concurrently developing mobile apps and wearable technology integrated with the Internet of Things to facilitate effective medication management for family members. In addition, research needs to expand the focus on social communication and education; popularize AD information to enhance public awareness; and focus on cultural appropriateness, ethical issues, and technical dependability in technology diffusion to assure equity and sustainability.

### Contribution and Limitations

#### Knowledge Contribution

The contributions of this study are primarily evident in two principal characteristics. First, this study systematically identified and synthesized key factors influencing medication adherence among Chinese patients with AD through an online Delphi study. By integrating multidisciplinary expert perspectives, the study provided an empirically grounded understanding of adherence-related challenges and established a conceptual foundation for future intervention development.

Second, this study focused on exploring the potential implications of these factors for future AI-supported medication management in a theoretical and exploratory manner rather than validating or implementing an AI-based system. By clarifying how adherence-related factors may inform the design of technology-assisted interventions, the study offers a preliminary framework to guide subsequent research aimed at supporting family caregivers and improving medication adherence among people with AD.

#### Research Limitations

This study elucidated the principal factors influencing medication adherence among Chinese patients with AD. It examined the prospective use of AI in medication management, establishing a theoretical basis for subsequent research in this domain. This study, however, possessed the following limitations. Owing to time limitations, only 12 participants completed the interviews, resulting in a limited sample size that may affect the generalizability of the findings. Second, the restricted representation of the core group, comprising 8 family members at various stages of the disease, may have complicated the data processing, potentially impacting the generalizability of the findings. Subsequent research should increase the sample size to better reflect family members and carers and refine the classification of patients by disease stage to augment the reliability and applicability of the results.

Moreover, although the panel size was sufficient for an exploratory Delphi study, there was a limited number of participants with direct AI implementation experience. Future studies should involve a broader range of specialists, such as neurologists, clinical pharmacists, and AI practitioners, to further validate and extend the findings. All of these will establish a more robust basis for an in-depth examination of the critical concerns in drug administration for people with AD.

### Conclusion

This study thoroughly examined the factors impacting medication adherence and the demands of Chinese patients with AD from several viewpoints. It further examined the domain of AI, emphasizing the primary impacting elements and requirements, uncovering prospects in precision therapy and educational support. Precision therapy is anticipated to facilitate individualized drug management by using technologies like ML and DL. Furthermore, AI-driven educational assistance can furnish comprehensive information to patients’ families and aid patients with making informed decisions. Although the potential applications of AI in medication management are vast, it is essential to address the issues of technological reliability, user experience, and ethical and societal ramifications.

## Supplementary material

10.2196/89508 Multimedia Appendix 1 Questions asked of reach respondent type.

10.2196/89508 Multimedia Appendix 2 Kappa analysis process.

10.2196/89508 Multimedia Appendix 3 Round 2 questionnaire.

10.2196/89508 Checklist 1 Delphi studies in social and health sciences – recommendations for an interdisciplinary, standardized reporting (Delphi-STAR).

## Abbreviations

AD: Alzheimer disease
AI: artificial intelligence
DL: deep learning
ML: machine learning
RQ: research question
