# Supplementary material for: Exploring Influencing Factors of Medication Adherence Among Chinese Patients With Alzheimer Disease: Delphi Study Informing Future Artificial Intelligence–Supported Interventions
Source: JMIR Form Res. 2026 Apr 17;10:e89508. doi: 10.2196/89508 (PMC13100466; doi:10.2196/89508)
Supplement: Multimedia Appendix 1 [file formative-v10-e89508-s001.docx]

| **Question** | **Participant** | | | |
| --- | --- | --- | --- | --- |
|  | Carer | Doctor | Other relevant  （Hospital and health care facility managers/  government health department officials/  leaders of medical research organizations/  drug company executives) | Company  designer |
| 1.What is your experience with patient medication? What do you know about this disease and medication for the disease? | √ | √ | √ |  |
| 2.What do you think is the role of the family in the patient's care? Or what aspects of the patient's medication do they need to give more attention to? | √ | √ | √ | √ |
| 3.What’s your experience with smart packaging (with your patients)? | √ | √ | √ | √ |
| 1. How patients is actually taking their prescribing treatments? What is their behaviour? |  | √ |  | √ |
| 5.How does the stage of the disease affect the patient's adherence behaviour? | √ | √ | √ |  |
| 6.How families supported patient in the adherence? What's the role of package? |  |  |  | √ |
| 7. How smart packaging can be improved to support AD patients adherence and what kind of feathers can help this support? |  | √ |  | √ |
| 8. What do you think about user patients' experience？How smart packaging can improve patient experience? |  |  |  | √ |
