# Supplementary material for: Exploring Influencing Factors of Medication Adherence Among Chinese Patients With Alzheimer Disease: Delphi Study Informing Future Artificial Intelligence–Supported Interventions
Source: JMIR Form Res. 2026 Apr 17;10:e89508. doi: 10.2196/89508 (PMC13100466; doi:10.2196/89508)
Supplement: Multimedia Appendix 2 [file formative-v10-e89508-s002.docx]

Kappa analysis process.

| **starting (point)** | **R1** | **R2** | **R1** | **R2** |
| --- | --- | --- | --- | --- |
| **Family and social support** | **Family and social support** | **Family and social support** | 1 | 1 |
| Family care and social support | Relatives' care and societal help | Family assistance | 1 | 1 |
| Emotional support | Psychological support | Mental help | 1 | 1 |
| Family environment | Family ambiance | Family atmosphere | 1 | 1 |
| Therapeutic assistance |  |  | 0 | 0 |
| Caregiver education | Family education |  | 1 | 0 |
| **Patient self-management** | **Patient self-management** | **Patient self-management** | 1 | 1 |
| Habits and behaviour | Patients’ behaviour | Patients’ behaviour | 1 | 1 |
| Cognitive decay | Memory decay | Cognitive decay | 1 | 1 |
| Treatment awareness | Treatment awareness | Treatment awareness | 1 | 1 |
| Complexity of medication regimens | Medication regimen intricacy | Complexity of medication regimens | 1 | 1 |
| Treatment motivation | Motivation about treatment | Treatment motivation | 1 | 1 |
| **Medical management challenges** | **Medical management challenges** | **Medical management challenges** | 1 | 1 |
| Doctor-patient communication | Doctor-patient communication |  | 1 | 0 |
| Progression of illness |  |  | 0 | 0 |
| Financial factors |  |  | 0 | 0 |
| Therapeutic effects | Therapeutic effects |  | 1 | 0 |
| Clinical progression |  |  | 0 | 0 |
| **Medical Service Design** | **Medical Service Design** | **Service Design** | 1 | 1 |
| Cultural awareness | Social awareness | Cultural awareness | 1 | 1 |
| Human-computer interaction | Interaction | Interaction with applications | 1 | 1 |
| User demands and experience | User experience | User experience | 1 | 1 |
| Assistive tools |  | Applications | 0 | 1 |
| Technology and innovation |  | Technology | 0 | 1 |
