# Supplementary material for: Exploring Influencing Factors of Medication Adherence Among Chinese Patients With Alzheimer Disease: Delphi Study Informing Future Artificial Intelligence–Supported Interventions
Source: JMIR Form Res. 2026 Apr 17;10:e89508. doi: 10.2196/89508 (PMC13100466; doi:10.2196/89508)
Supplement: Multimedia Appendix 3 [file formative-v10-e89508-s003.docx]

**Round 2 Questionnaire**

**Survey on Factors Influencing Medication Adherence in Patients with Alzheimer’s Disease**

This study investigates the factors influencing medication adherence among patients with Alzheimer’s disease.
Please read the explanation of each factor before selecting the level of influence.

**Medication adherence** refers to the extent to which a patient accepts, agrees with, and correctly follows a prescribed treatment plan. This includes taking medication at the correct time and dosage, attending follow-up appointments, and adhering to any dietary restrictions related to the medication. Simply put, it means following the doctor’s instructions as prescribed.

Please indicate how much you think each factor affects medication adherence in patients with Alzheimer’s disease.
**[Single choice question, scale 1–5, where 5 = very strong influence and 1 = very little influence]**

**1. Cognitive Decline**
Mainly refers to memory impairment, reduced executive function, and deteriorating recall ability.
How much do you think cognitive decline affects medication adherence?

○5 ○4 ○3 ○2 ○1

**2. Awareness of Treatment**
Lack of awareness may lead to early resistance to medication and poor recognition of the need for treatment.
How much do you think awareness of treatment affects medication adherence?

○5 ○4 ○3 ○2 ○1

**3. Treatment Motivation**
Includes mental health status, beliefs and attitudes toward treatment, and active participation in the treatment process.
How much do you think treatment motivation affects medication adherence?

○5 ○4 ○3 ○2 ○1

**4. Complexity of the Treatment Plan**
Involves polypharmacy, frequent dosing schedules, difficulty tracking dosage, and non-oral routes.
How much do you think complexity of the treatment plan affects medication adherence?

○5 ○4 ○3 ○2 ○1

**5. Patient Habits and Behaviors**
Refers to harmful personal habits such as smoking and alcohol use.
How much do you think patient habits and behaviors affect medication adherence?

○5 ○4 ○3 ○2 ○1

**6. Family and Social Support**
Includes care from family and community members, such as home-based care.
How much do you think family and social support affects medication adherence?

○5 ○4 ○3 ○2 ○1

**7. Emotional Support**
Includes companionship, positive attitudes toward treatment, and emotional encouragement.
How much do you think emotional support affects medication adherence?

○5 ○4 ○3 ○2 ○1

**8. Treatment Assistance**
Refers to help with medication management, monitoring from family, and communication with healthcare providers.
How much do you think treatment assistance affects medication adherence?

○5 ○4 ○3 ○2 ○1

**9. Caregiver’s Professional Education**
Includes caregiving skills, communication abilities, stress management, and understanding of the disease and medications.
How much do you think caregiver education affects medication adherence?

○5 ○4 ○3 ○2 ○1

**10. Family Environment**
Includes family stability, willingness to support treatment, whether the patient lives alone, and overall living conditions.
How much do you think the family environment affects medication adherence?

○5 ○4 ○3 ○2 ○1

**11. Disease Progression**
Includes the stage of the disease, worsening symptoms, changes in treatment plans, and duration of treatment.
How much do you think disease progression affects medication adherence?

○5 ○4 ○3 ○2 ○1

**12. Clinical Developments**
Includes overall treatment strategies and types of medication used.
How much do you think clinical developments affect medication adherence?

○5 ○4 ○3 ○2 ○1

**13. Treatment Effectiveness**
Covers side effects, efficacy, delayed effects, and dosage accuracy.
How much do you think treatment effectiveness affects medication adherence?

○5 ○4 ○3 ○2 ○1

**14. Doctor-Patient Communication**
Refers to communication between caregivers and healthcare providers.
How much do you think doctor-patient communication affects medication adherence?

○5 ○4 ○3 ○2 ○1

**15. Economic Factors**
Includes treatment costs and financial burden.
How much do you think economic factors affect medication adherence?

○5 ○4 ○3 ○2 ○1

**16. Assistive Tools**
Includes smart tools such as pill boxes, compartment dividers, and intelligent packaging.
How much do you think assistive tools affect medication adherence?

○5 ○4 ○3 ○2 ○1

**17. Technology and Innovation**
Includes useful technologies such as AI, AR, and image processing.
How much do you think technology and innovation affect medication adherence?

○5 ○4 ○3 ○2 ○1

**18. User Needs and Experience**
Refers to accessible design, clarity of information, and ease of use.
How much do you think user needs and experience affect medication adherence?

○5 ○4 ○3 ○2 ○1

**19. Interactive Features and Formats**
Includes clear packaging and labeling, support platforms, flexible healthcare access, and medication management apps.
How much do you think interactive features and formats affect medication adherence?

○5 ○4 ○3 ○2 ○1

**20. Cultural Ideologies**
Covers medication beliefs, communication styles, openness to new products, and cultural norms (e.g., filial piety and hands-on caregiving).
How much do you think cultural ideologies affect medication adherence?

○5 ○4 ○3 ○2 ○1

Round 3 Questionnaire

N1：

**Title: Survey on Factors Influencing Medication Adherence in Patients with Alzheimer’s Disease**

Please review the explanations of each factor and reconsider your previous selection based on the average rating in the previous round.

**Medication adherence** refers to a patient’s ability to accept, agree with, and properly follow a treatment plan, including accurate medication timing and dosage, follow-up visits, and dietary restrictions when applicable.

Please confirm your rating for each of the following items.
**[Single choice question: 5 = very strong influence, 1 = very little influence]**

**1. Cognitive Decline**
Mainly refers to memory loss, impaired recall, and reduced executive function.
*The average score in the previous round was 3.88. Your previous rating was 5. Please confirm your selection.*
How much do you think cognitive decline affects medication adherence?

○5 ○4 ○3 ○2 ○1

**2. Awareness of Treatment**
Patients without treatment awareness may initially resist medication and lack understanding of treatment needs.
*The average score in the previous round was 3.88. Your previous rating was 5. Please confirm your selection.*
How much do you think treatment awareness affects medication adherence?

○5 ○4 ○3 ○2 ○1

**3. Treatment Motivation**
Includes psychological health, treatment beliefs and attitudes, and active participation in care.
*The average score in the previous round was 3.75. Your previous rating was 1. Please confirm your selection.*
How much do you think treatment motivation affects medication adherence?

○5 ○4 ○3 ○2 ○1

**4. Complexity of the Treatment Plan**
Includes polypharmacy, complex dosing schedules, difficult dosage tracking, and non-oral administration.
*The average score in the previous round was 3.25. Your previous rating was 5. Please confirm your selection.*
How much do you think treatment plan complexity affects medication adherence?

○5 ○4 ○3 ○2 ○1

**5. Patient Habits and Behaviors**
Such as smoking, alcohol consumption, or other harmful habits.
*The average score in the previous round was 4.25. Your previous rating was 5. Please confirm your selection.*
How much do you think patient habits and behaviors affect medication adherence?

○5 ○4 ○3 ○2 ○1

**6. Family and Social Support**
Includes home-based care and support from family or community members.
*The average score in the previous round was 4.75. Your previous rating was 5. Please confirm your selection.*
How much do you think family and social support affects medication adherence?

○5 ○4 ○3 ○2 ○1

**7. Emotional Support**
Includes family companionship, encouragement, and emotional understanding.
*The average score in the previous round was 4.25. Your previous rating was 2. Please confirm your selection.*
How much do you think emotional support affects medication adherence?

○5 ○4 ○3 ○2 ○1

**8. Treatment Assistance**
Includes family monitoring, medication management help, and communication with healthcare providers.
*The average score in the previous round was 4.38. Your previous rating was 4. Please confirm your selection.*
How much do you think treatment assistance affects medication adherence?

○5 ○4 ○3 ○2 ○1

**9. Caregiver’s Professional Education**
Covers caregiving skills, effective communication, reduced caregiver burden, and understanding of Alzheimer’s disease and medication.
*The average score in the previous round was 4.38. Your previous rating was 2. Please confirm your selection.*
How much do you think caregiver education affects medication adherence?

○5 ○4 ○3 ○2 ○1

**10. Family Environment**
Includes family stability, cooperation with treatment, whether the patient lives alone, and general living conditions.
*The average score in the previous round was 4.63. Your previous rating was 5. Please confirm your selection.*
How much do you think family environment affects medication adherence?

○5 ○4 ○3 ○2 ○1

**11. Disease Progression**
Includes adherence at different disease stages, cognitive decline, worsening symptoms, and treatment plan changes.
*The average score in the previous round was 4.00. Your previous rating was 5. Please confirm your selection.*
How much do you think disease progression affects medication adherence?

○5 ○4 ○3 ○2 ○1

**12. Clinical Developments**
Includes overall treatment strategies and types of medications used.
*The average score in the previous round was 3.25. Your previous rating was 2. Please confirm your selection.*
How much do you think clinical developments affect medication adherence?

○5 ○4 ○3 ○2 ○1

**13. Treatment Effectiveness**
Includes side effects, efficacy, delayed drug effects, and precise dosing.
*The average score in the previous round was 3.5. Your previous rating was 4. Please confirm your selection.*
How much do you think treatment effectiveness affects medication adherence?

○5 ○4 ○3 ○2 ○1

**14. Doctor-Patient Communication**
Refers to interactions between the patient’s family and healthcare providers.
*The average score in the previous round was 3.75. Your previous rating was 4. Please confirm your selection.*
How much do you think doctor-patient communication affects medication adherence?

○5 ○4 ○3 ○2 ○1

**15. Economic Factors**
Includes treatment costs and financial burden.
*The average score in the previous round was 3.5. Your previous rating was 2. Please confirm your selection.*
How much do you think economic factors affect medication adherence?

○5 ○4 ○3 ○2 ○1

**16. Assistive Tools**
Refers to smart tools such as pill box dividers and intelligent packaging.
*The average score in the previous round was 3.25. Your previous rating was 3. Please confirm your selection.*
How much do you think assistive tools affect medication adherence?

○5 ○4 ○3 ○2 ○1

**17. Technology and Innovation**
Includes useful technologies such as artificial intelligence, AR, and image processing.
*The average score in the previous round was 3.25. Your previous rating was 3. Please confirm your selection.*
How much do you think technology and innovation affect medication adherence?

○5 ○4 ○3 ○2 ○1

**18. User Needs and Experience**
Includes accessible design, clarity of information, and ease of use.
*The average score in the previous round was 3.5. Your previous rating was 3. Please confirm your selection.*
How much do you think user needs and experience affect medication adherence?

○5 ○4 ○3 ○2 ○1

**19. Interactive Features and Formats**
Includes clear labeling, social support platforms, flexible healthcare access, and medication management apps.
*The average score in the previous round was 3.63. Your previous rating was 3. Please confirm your selection.*
How much do you think interactive features and formats affect medication adherence?

○5 ○4 ○3 ○2 ○1

**20. Cultural Ideologies**
Refers to beliefs about medication, communication styles, openness to new products, and cultural values such as filial piety and hands-on caregiving.
*The average score in the previous round was 3.75. Your previous rating was 5. Please confirm your selection.*
How much do you think cultural ideologies affect medication adherence?

○5 ○4 ○3 ○2 ○1

N2：

**Survey on Factors Influencing Medication Adherence in Patients with Alzheimer’s Disease**

Please review the explanation of each factor. You will also see the average score from the previous round and your own previous selection. Kindly confirm your rating again.

**[Single choice question: 5 = Very strong influence, 1 = Very little influence]**

**1. Cognitive Decline**
Mainly manifested as memory loss, impaired recall, and weakened executive function.
*The average score in the previous round was 3.88. Your previous rating was 3. Please confirm your selection.*

How much do you think cognitive decline affects medication adherence?
○5 ○4 ○3 ○2 ○1

**2. Awareness of Treatment**
Without awareness, patients may resist medication in the early stages and lack understanding of the need for treatment.
*The average score in the previous round was 3.88. Your previous rating was 4. Please confirm your selection.*

How much do you think awareness of treatment affects medication adherence?
○5 ○4 ○3 ○2 ○1

**3. Treatment Motivation**
Includes mental health, belief and attitude toward treatment, and level of engagement.
*The average score in the previous round was 3.75. Your previous rating was 2. Please confirm your selection.*

How much do you think treatment motivation affects medication adherence?
○5 ○4 ○3 ○2 ○1

**4. Complexity of Treatment Plan**
Involves polypharmacy, complex dosing schedules, difficulty in tracking each dose, and non-oral routes.
*The average score in the previous round was 3.25. Your previous rating was 4. Please confirm your selection.*

How much do you think treatment plan complexity affects medication adherence?
○5 ○4 ○3 ○2 ○1

**5. Patient Habits and Behaviors**
Includes harmful habits such as smoking and drinking alcohol.
*The average score in the previous round was 4.25. Your previous rating was 5. Please confirm your selection.*

How much do you think patient habits and behaviors affect medication adherence?
○5 ○4 ○3 ○2 ○1

**6. Family and Social Support**
Includes care from family and society, such as home caregiving.
*The average score in the previous round was 4.75. Your previous rating was 5. Please confirm your selection.*

How much do you think family and social support affects medication adherence?
○5 ○4 ○3 ○2 ○1

**7. Emotional Support**
Includes companionship, supportive treatment attitude, emotional understanding (e.g., encouragement).
*The average score in the previous round was 4.25. Your previous rating was 2. Please confirm your selection.*

How much do you think emotional support affects medication adherence?
○5 ○4 ○3 ○2 ○1

**8. Treatment Assistance**
Includes family monitoring of medication, support in medication management, and communication with healthcare providers.
*The average score in the previous round was 4.38. Your previous rating was 4. Please confirm your selection.*

How much do you think treatment assistance affects medication adherence?
○5 ○4 ○3 ○2 ○1

**9. Caregiver’s Professional Education**
Refers to caregiving skills, effective communication, psychological burden relief, and understanding of the condition and medications.
*The average score in the previous round was 4.38. Your previous rating was 3. Please confirm your selection.*

How much do you think caregiver’s education affects medication adherence?
○5 ○4 ○3 ○2 ○1

**10. Family Environment**
Includes family stability, level of cooperation in treatment, whether the patient lives alone, and living conditions.
*The average score in the previous round was 4.63. Your previous rating was 5. Please confirm your selection.*

How much do you think family environment affects medication adherence?
○5 ○4 ○3 ○2 ○1

**11. Disease Progression**
Includes adherence at different stages, cognitive decline, worsening symptoms, changes in treatment plans, and duration of treatment.
*The average score in the previous round was 4.00. Your previous rating was 4. Please confirm your selection.*

How much do you think disease progression affects medication adherence?
○5 ○4 ○3 ○2 ○1

**12. Clinical Developments**
Includes treatment strategies such as combination therapy and drug types.
*The average score in the previous round was 3.25. Your previous rating was 4. Please confirm your selection.*

How much do you think clinical developments affect medication adherence?
○5 ○4 ○3 ○2 ○1

**13. Treatment Effectiveness**
Includes side effects, drug efficacy, delayed effects, and accurate dosing.
*The average score in the previous round was 3.50. Your previous rating was 1. Please confirm your selection.*

How much do you think treatment effectiveness affects medication adherence?
○5 ○4 ○3 ○2 ○1

**14. Doctor-Patient Communication**
Refers to communication between the patient’s family and the healthcare provider.
*The average score in the previous round was 3.75. Your previous rating was 2. Please confirm your selection.*

How much do you think doctor-patient communication affects medication adherence?
○5 ○4 ○3 ○2 ○1

**15. Economic Factors**
Includes treatment costs and financial burden.
*The average score in the previous round was 3.50. Your previous rating was 2. Please confirm your selection.*

How much do you think economic factors affect medication adherence?
○5 ○4 ○3 ○2 ○1

**16. Assistive Tools**
Refers to digital tools such as pill box dividers or smart packaging.
*The average score in the previous round was 3.25. Your previous rating was 3. Please confirm your selection.*

How much do you think assistive tools affect medication adherence?
○5 ○4 ○3 ○2 ○1

**17. Technology and Innovation**
Includes technologies like artificial intelligence, augmented reality, image processing, etc.
*The average score in the previous round was 3.25. Your previous rating was 4. Please confirm your selection.*

How much do you think technology and innovation affect medication adherence?
○5 ○4 ○3 ○2 ○1

**18. User Needs and Experience**
Refers to accessibility, clarity of information, and user-friendly operation.
*The average score in the previous round was 3.50. Your previous rating was 3. Please confirm your selection.*

How much do you think user needs and experience affect medication adherence?
○5 ○4 ○3 ○2 ○1

**19. Interactive Features and Formats**
Includes clear labeling, support platforms, flexible healthcare access, and medication management apps (e.g., supervision, filtering information).
*The average score in the previous round was 3.63. Your previous rating was 4. Please confirm your selection.*

How much do you think interactive features and formats affect medication adherence?
○5 ○4 ○3 ○2 ○1

**20. Cultural Ideologies**
Refers to beliefs about medication use, communication preferences, openness to new products, and cultural factors (e.g., filial piety, hands-on caregiving).
*The average score in the previous round was 3.75. Your previous rating was 3. Please confirm your selection.*

How much do you think cultural ideologies affect medication adherence?
○5 ○4 ○3 ○2 ○1

N3：

**Survey on Factors Influencing Medication Adherence in Patients with Alzheimer’s Disease**

Please review each of the following factors. You will see the average score from the previous round and your previous selection. Kindly confirm your rating.

**[Single-choice question: 5 = Very strong influence, 1 = Very little influence]**

**1. Cognitive Decline**
Mainly refers to memory loss, impaired recall, and reduced executive function.
*Average score from the previous round: 3.88. Your previous rating: 4. Please confirm your selection.*
How much do you think cognitive decline affects medication adherence?

○5 ○4 ○3 ○2 ○1

**2. Awareness of Treatment**
Without treatment awareness, patients may easily resist medication in the early stages.
*Average score: 3.88. Your previous rating: 5. Please confirm your selection.*
How much do you think awareness of treatment affects medication adherence?

○5 ○4 ○3 ○2 ○1

**3. Treatment Motivation**
Includes the patient’s mental health, beliefs and attitudes toward treatment, and level of engagement.
*Average score: 3.75. Your previous rating: 5. Please confirm your selection.*
How much do you think treatment motivation affects medication adherence?

○5 ○4 ○3 ○2 ○1

**4. Complexity of the Treatment Plan**
Includes polypharmacy, complex dosage frequency, difficulty in tracking each dosage, and non-oral medication routes.
*Average score: 3.25. Your previous rating: 5. Please confirm your selection.*
How much do you think treatment complexity affects medication adherence?

○5 ○4 ○3 ○2 ○1

**5. Patient Habits and Behaviors**
Refers to harmful personal habits, such as smoking and drinking.
*Average score: 4.25. Your previous rating: 4. Please confirm your selection.*
How much do you think patient habits and behaviors affect medication adherence?

○5 ○4 ○3 ○2 ○1

**6. Family and Social Support**
Includes caregiving support from family and the community (e.g., home care).
*Average score: 4.75. Your previous rating: 5. Please confirm your selection.*
How much do you think family and social support affects medication adherence?

○5 ○4 ○3 ○2 ○1

**7. Emotional Support**
Includes companionship, encouragement, and emotional understanding.
*Average score: 4.25. Your previous rating: 5. Please confirm your selection.*
How much do you think emotional support affects medication adherence?

○5 ○4 ○3 ○2 ○1

**8. Treatment Assistance**
Refers to family assistance with medication monitoring and management, and communication with healthcare professionals.
*Average score: 4.38. Your previous rating: 5. Please confirm your selection.*
How much do you think treatment assistance affects medication adherence?

○5 ○4 ○3 ○2 ○1

**9. Caregiver’s Professional Education**
Includes caregiving skills, communication effectiveness, stress management, and understanding of Alzheimer’s disease and medications.
*Average score: 4.38. Your previous rating: 5. Please confirm your selection.*
How much do you think caregiver education affects medication adherence?

○5 ○4 ○3 ○2 ○1

**10. Family Environment**
Includes household stability, cooperation in treatment, whether the patient lives alone, and overall living conditions.
*Average score: 4.63. Your previous rating: 5. Please confirm your selection.*
How much do you think family environment affects medication adherence?

○5 ○4 ○3 ○2 ○1

**11. Disease Progression**
Includes adherence across disease stages, cognitive deterioration, worsening conditions, and treatment plan changes.
*Average score: 4.00. Your previous rating: 4. Please confirm your selection.*
How much do you think disease progression affects medication adherence?

○5 ○4 ○3 ○2 ○1

**12. Clinical Developments**
Includes treatment strategies, combination therapies, and types of medications.
*Average score: 3.25. Your previous rating: 3. Please confirm your selection.*
How much do you think clinical developments affect medication adherence?

○5 ○4 ○3 ○2 ○1

**13. Treatment Effectiveness**
Includes drug side effects, therapeutic efficacy, delayed effects, and dose accuracy.
*Average score: 3.5. Your previous rating: 3. Please confirm your selection.*
How much do you think treatment effectiveness affects medication adherence?

○5 ○4 ○3 ○2 ○1

**14. Doctor–Patient Communication**
Refers to communication between caregivers and healthcare providers.
*Average score: 3.75. Your previous rating: 4. Please confirm your selection.*
How much do you think doctor–patient communication affects medication adherence?

○5 ○4 ○3 ○2 ○1

**15. Economic Factors**
Includes the cost of treatment and overall financial burden.
*Average score: 3.5. Your previous rating: 5. Please confirm your selection.*
How much do you think economic factors affect medication adherence?

○5 ○4 ○3 ○2 ○1

**16. Assistive Tools**
Includes smart tools such as pill organizers, dividers, and intelligent packaging.
*Average score: 3.25. Your previous rating: 4. Please confirm your selection.*
How much do you think assistive tools affect medication adherence?

○5 ○4 ○3 ○2 ○1

**17. Technology and Innovation**
Refers to effective technologies such as artificial intelligence, AR, image processing, etc.
*Average score: 3.25. Your previous rating: 5. Please confirm your selection.*
How much do you think technology and innovation affect medication adherence?

○5 ○4 ○3 ○2 ○1

**18. User Needs and Experience**
Includes accessible design, clarity of information, and ease of operation.
*Average score: 3.5. Your previous rating: 5. Please confirm your selection.*
How much do you think user needs and experience affect medication adherence?

○5 ○4 ○3 ○2 ○1

**19. Interactive Features and Formats**
Includes clear packaging, social support platforms, accessibility and flexibility of healthcare services, and medication management apps (e.g., supervision, filtered information).
*Average score: 3.63. Your previous rating: 4. Please confirm your selection.*
How much do you think interactive features and formats affect medication adherence?

○5 ○4 ○3 ○2 ○1

**20. Cultural Ideologies**
Includes beliefs about medication, communication style, openness to new products, and cultural factors (e.g., filial piety and hands-on caregiving).
*Average score: 3.75. Your previous rating: 5. Please confirm your selection.*
How much do you think cultural ideologies affect medication adherence?

○5 ○4 ○3 ○2 ○1

N4：

**Survey on Factors Influencing Medication Adherence in Patients with Alzheimer’s Disease**

Please review the description of each factor. The average score from the previous round and your previous selection are shown for reference. Kindly reconfirm your choice.

**[Single-choice question: 5 = Very strong influence, 1 = Very little influence]**

**1. Cognitive Decline**
Mainly manifested as memory loss, impaired recall, and reduced executive function.
*Average score from the previous round: 3.88. Your previous rating: 3. Please confirm your selection.*
How much do you think cognitive decline affects medication adherence?

○5 ○4 ○3 ○2 ○1

**2. Awareness of Treatment**
Patients lacking treatment awareness may resist medication in early stages.
*Average score: 3.88. Your previous rating: 3. Please confirm your selection.*
How much do you think awareness of treatment affects medication adherence?

○5 ○4 ○3 ○2 ○1

**3. Treatment Motivation**
Includes mental health, beliefs and attitudes about treatment, and active engagement in therapy.
*Average score: 3.75. Your previous rating: 3. Please confirm your selection.*
How much do you think treatment motivation affects medication adherence?

○5 ○4 ○3 ○2 ○1

**4. Complexity of the Treatment Plan**
Refers to polypharmacy, complex dosing frequency, difficulty in dose tracking, and non-oral routes.
*Average score: 3.25. Your previous rating: 4. Please confirm your selection.*
How much do you think treatment complexity affects medication adherence?

○5 ○4 ○3 ○2 ○1

**5. Patient Habits and Behaviors**
Includes harmful personal habits, such as smoking and drinking alcohol.
*Average score: 4.25. Your previous rating: 5. Please confirm your selection.*
How much do you think patient habits and behaviors affect medication adherence?

○5 ○4 ○3 ○2 ○1

**6. Family and Social Support**
Refers to care provided by family or community, such as home care services.
*Average score: 4.75. Your previous rating: 5. Please confirm your selection.*
How much do you think family and social support affects medication adherence?

○5 ○4 ○3 ○2 ○1

**7. Emotional Support**
Includes companionship, emotional understanding, and encouragement.
*Average score: 4.25. Your previous rating: 5. Please confirm your selection.*
How much do you think emotional support affects medication adherence?

○5 ○4 ○3 ○2 ○1

**8. Treatment Assistance**
Includes family help in medication monitoring, management, and communication with healthcare providers.
*Average score: 4.38. Your previous rating: 4. Please confirm your selection.*
How much do you think treatment assistance affects medication adherence?

○5 ○4 ○3 ○2 ○1

**9. Caregiver’s Professional Education**
Includes skills in caregiving, communication, stress management, and understanding of Alzheimer’s and medications.
*Average score: 4.38. Your previous rating: 5. Please confirm your selection.*
How much do you think caregiver education affects medication adherence?

○5 ○4 ○3 ○2 ○1

**10. Family Environment**
Includes family stability, cooperation in treatment, whether the patient lives alone, and living conditions.
*Average score: 4.63. Your previous rating: 4. Please confirm your selection.*
How much do you think family environment affects medication adherence?

○5 ○4 ○3 ○2 ○1

**11. Disease Progression**
Covers adherence changes during disease stages, cognitive decline, worsening condition, treatment changes, and duration.
*Average score: 4.00. Your previous rating: 5. Please confirm your selection.*
How much do you think disease progression affects medication adherence?

○5 ○4 ○3 ○2 ○1

**12. Clinical Developments**
Refers to treatment strategies, such as drug combinations and types of medication.
*Average score: 3.25. Your previous rating: 3. Please confirm your selection.*
How much do you think clinical developments affect medication adherence?

○5 ○4 ○3 ○2 ○1

**13. Treatment Effectiveness**
Includes side effects, therapeutic impact, delayed drug response, and accurate dosage.
*Average score: 3.5. Your previous rating: 5. Please confirm your selection.*
How much do you think treatment effectiveness affects medication adherence?

○5 ○4 ○3 ○2 ○1

**14. Doctor–Patient Communication**
Refers to communication between caregivers/family and healthcare professionals.
*Average score: 3.75. Your previous rating: 5. Please confirm your selection.*
How much do you think doctor–patient communication affects medication adherence?

○5 ○4 ○3 ○2 ○1

**15. Economic Factors**
Includes the cost of treatment and financial burden on the patient or family.
*Average score: 3.5. Your previous rating: 3. Please confirm your selection.*
How much do you think economic factors affect medication adherence?

○5 ○4 ○3 ○2 ○1

**16. Assistive Tools**
Refers to smart aids such as pill organizers, compartment boxes, or smart packaging.
*Average score: 3.25. Your previous rating: 5. Please confirm your selection.*
How much do you think assistive tools affect medication adherence?

○5 ○4 ○3 ○2 ○1

**17. Technology and Innovation**
Includes useful technologies such as artificial intelligence, augmented reality (AR), and image processing.
*Average score: 3.25. Your previous rating: 3. Please confirm your selection.*
How much do you think technology and innovation affect medication adherence?

○5 ○4 ○3 ○2 ○1

**18. User Needs and Experience**
Refers to accessibility, clarity of information, and ease of use.
*Average score: 3.5. Your previous rating: 5. Please confirm your selection.*
How much do you think user needs and experience affect medication adherence?

○5 ○4 ○3 ○2 ○1

**19. Interactive Features and Formats**
Includes clear labeling and packaging, social support platforms, healthcare access, flexibility, and medication apps (e.g., for supervision or filtering information).
*Average score: 3.63. Your previous rating: 5. Please confirm your selection.*
How much do you think interactive features and formats affect medication adherence?

○5 ○4 ○3 ○2 ○1

**20. Cultural Ideologies**
Refers to beliefs about medication management, communication styles, acceptance of new products, and cultural values (e.g., filial piety and hands-on caregiving).
*Average score: 3.75. Your previous rating: 4. Please confirm your selection.*
How much do you think cultural ideologies affect medication adherence?

○5 ○4 ○3 ○2 ○1

N5：

**Survey on Factors Influencing Medication Adherence in Patients with Alzheimer’s Disease**

Please review the explanation of each factor. The average score from the previous round and your own previous selection are shown for reference. Kindly confirm your selection again.

**[Single-choice question: 5 = Very strong influence, 1 = Very little influence]**

**1. Cognitive Decline**
Mainly manifested as memory loss, impaired recall, and reduced executive functioning.
*Average score from the previous round: 3.88. Your previous rating: 5. Please confirm your selection.*
How much do you think cognitive decline affects medication adherence?

○5 ○4 ○3 ○2 ○1

**2. Awareness of Treatment**
Patients lacking awareness may resist medication in early stages and fail to recognize the need for treatment.
*Average score: 3.88. Your previous rating: 3. Please confirm your selection.*
How much do you think awareness of treatment affects medication adherence?

○5 ○4 ○3 ○2 ○1

**3. Treatment Motivation**
Includes psychological well-being, treatment beliefs and attitudes, and active participation in care.
*Average score: 3.75. Your previous rating: 4. Please confirm your selection.*
How much do you think treatment motivation affects medication adherence?

○5 ○4 ○3 ○2 ○1

**4. Complexity of the Treatment Plan**
Includes polypharmacy, complex dosing frequency, difficulty tracking medication, and non-oral administration routes.
*Average score: 3.25. Your previous rating: 2. Please confirm your selection.*
How much do you think treatment complexity affects medication adherence?

○5 ○4 ○3 ○2 ○1

**5. Patient Habits and Behaviors**
Refers to harmful personal habits, such as smoking and alcohol consumption.
*Average score: 4.25. Your previous rating: 5. Please confirm your selection.*
How much do you think patient habits and behaviors affect medication adherence?

○5 ○4 ○3 ○2 ○1

**6. Family and Social Support**
Includes caregiving provided by family or community, such as home care.
*Average score: 4.75. Your previous rating: 5. Please confirm your selection.*
How much do you think family and social support affects medication adherence?

○5 ○4 ○3 ○2 ○1

**7. Emotional Support**
Includes companionship, encouragement, emotional understanding, and positive treatment attitude.
*Average score: 4.25. Your previous rating: 5. Please confirm your selection.*
How much do you think emotional support affects medication adherence?

○5 ○4 ○3 ○2 ○1

**8. Treatment Assistance**
Includes medication supervision by family, help with managing medication routines, and communication with doctors.
*Average score: 4.38. Your previous rating: 5. Please confirm your selection.*
How much do you think treatment assistance affects medication adherence?

○5 ○4 ○3 ○2 ○1

**9. Caregiver’s Professional Education**
Includes caregiving skills, effective communication, psychological stress relief, and knowledge of Alzheimer's and medications.
*Average score: 4.38. Your previous rating: 5. Please confirm your selection.*
How much do you think caregiver education affects medication adherence?

○5 ○4 ○3 ○2 ○1

**10. Family Environment**
Includes family stability, willingness to cooperate with treatment, whether the patient lives alone, and living conditions.
*Average score: 4.63. Your previous rating: 5. Please confirm your selection.*
How much do you think the family environment affects medication adherence?

○5 ○4 ○3 ○2 ○1

**11. Disease Progression**
Covers adherence across disease stages, cognitive deterioration, worsening symptoms, changes in treatment plans, and treatment duration.
*Average score: 4.00. Your previous rating: 5. Please confirm your selection.*
How much do you think disease progression affects medication adherence?

○5 ○4 ○3 ○2 ○1

**12. Clinical Developments**
Includes treatment strategies such as combination therapy and different medication types.
*Average score: 3.25. Your previous rating: 3. Please confirm your selection.*
How much do you think clinical developments affect medication adherence?

○5 ○4 ○3 ○2 ○1

**13. Treatment Effectiveness**
Includes side effects, drug efficacy, delayed drug response, and dosage accuracy.
*Average score: 3.5. Your previous rating: 3. Please confirm your selection.*
How much do you think treatment effectiveness affects medication adherence?

○5 ○4 ○3 ○2 ○1

**14. Doctor–Patient Communication**
Refers to communication between caregivers/family members and healthcare professionals.
*Average score: 3.75. Your previous rating: 3. Please confirm your selection.*
How much do you think doctor–patient communication affects medication adherence?

○5 ○4 ○3 ○2 ○1

**15. Economic Factors**
Includes treatment costs and overall financial burden.
*Average score: 3.5. Your previous rating: 2. Please confirm your selection.*
How much do you think economic factors affect medication adherence?

○5 ○4 ○3 ○2 ○1

**16. Assistive Tools**
Includes smart applications such as pill organizers, compartment boxes, and intelligent packaging.
*Average score: 3.25. Your previous rating: 1. Please confirm your selection.*
How much do you think assistive tools affect medication adherence?

○5 ○4 ○3 ○2 ○1

**17. Technology and Innovation**
Includes useful technologies like artificial intelligence, augmented reality (AR), and image processing.
*Average score: 3.25. Your previous rating: 2. Please confirm your selection.*
How much do you think technology and innovation affect medication adherence?

○5 ○4 ○3 ○2 ○1

**18. User Needs and Experience**
Refers to accessibility, clear information delivery, ease of understanding, and user-friendly operations.
*Average score: 3.5. Your previous rating: 2. Please confirm your selection.*
How much do you think user needs and experience affect medication adherence?

○5 ○4 ○3 ○2 ○1

**19. Interactive Features and Formats**
Includes clear labeling and packaging, social support platforms, accessible and flexible healthcare, and medication management apps (e.g., supervision, filtering information).
*Average score: 3.63. Your previous rating: 2. Please confirm your selection.*
How much do you think interactive features and formats affect medication adherence?

○5 ○4 ○3 ○2 ○1

**20. Cultural Ideologies**
Includes beliefs about medication use, communication styles, openness to new products, and cultural values such as filial piety and hands-on caregiving.
*Average score: 3.75. Your previous rating: 4. Please confirm your selection.*
How much do you think cultural ideologies affect medication adherence?

○5 ○4 ○3 ○2 ○1

N6：

**Survey on Factors Influencing Medication Adherence in Patients with Alzheimer’s Disease**

Please review the explanation of each factor. You will find the average rating from the previous round and your own previous response. Kindly confirm your selection again.

**[Single-choice question: 5 = Very strong influence, 1 = Very little influence]**

**1. Cognitive Decline**
Mainly manifested as memory loss, impaired recall, and reduced executive function.
*Average score from the previous round: 3.88. Your previous rating: 2. Please confirm your selection.*
How much do you think cognitive decline affects medication adherence?

○5 ○4 ○3 ○2 ○1

**2. Awareness of Treatment**
Patients with no treatment awareness may resist medication in the early stages.
*Average score: 3.88. Your previous rating: 4. Please confirm your selection.*
How much do you think awareness of treatment affects medication adherence?

○5 ○4 ○3 ○2 ○1

**3. Treatment Motivation**
Includes psychological health, treatment beliefs and attitudes, and level of active participation.
*Average score: 3.75. Your previous rating: 3. Please confirm your selection.*
How much do you think treatment motivation affects medication adherence?

○5 ○4 ○3 ○2 ○1

**4. Complexity of the Treatment Plan**
Includes polypharmacy, complex dosing frequency, difficulty in dosage tracking, and non-oral administration.
*Average score: 3.25. Your previous rating: 4. Please confirm your selection.*
How much do you think treatment plan complexity affects medication adherence?

○5 ○4 ○3 ○2 ○1

**5. Patient Habits and Behaviors**
Includes harmful habits such as smoking and drinking alcohol.
*Average score: 4.25. Your previous rating: 4. Please confirm your selection.*
How much do you think patient habits and behaviors affect medication adherence?

○5 ○4 ○3 ○2 ○1

**6. Family and Social Support**
Refers to care and support from family and society, such as home care.
*Average score: 4.75. Your previous rating: 4. Please confirm your selection.*
How much do you think family and social support affects medication adherence?

○5 ○4 ○3 ○2 ○1

**7. Emotional Support**
Includes companionship, treatment attitude, emotional understanding, and encouragement.
*Average score: 4.25. Your previous rating: 3. Please confirm your selection.*
How much do you think emotional support affects medication adherence?

○5 ○4 ○3 ○2 ○1

**8. Treatment Assistance**
Includes supervision of medication by family, help with medication management, and doctor-patient communication.
*Average score: 4.38. Your previous rating: 3. Please confirm your selection.*
How much do you think treatment assistance affects medication adherence?

○5 ○4 ○3 ○2 ○1

**9. Caregiver’s Professional Education**
Refers to caregiving skills, effective communication, stress relief, and understanding of Alzheimer’s and related medications.
*Average score: 4.38. Your previous rating: 3. Please confirm your selection.*
How much do you think caregiver education affects medication adherence?

○5 ○4 ○3 ○2 ○1

**10. Family Environment**
Includes family stability, cooperation in treatment, whether the patient lives alone, and general living conditions.
*Average score: 4.63. Your previous rating: 4. Please confirm your selection.*
How much do you think family environment affects medication adherence?

○5 ○4 ○3 ○2 ○1

**11. Disease Progression**
Covers adherence at different stages, cognitive decline, worsening symptoms, changes in treatment, and treatment duration.
*Average score: 4.00. Your previous rating: 3. Please confirm your selection.*
How much do you think disease progression affects medication adherence?

○5 ○4 ○3 ○2 ○1

**12. Clinical Developments**
Includes treatment strategies such as combination therapy and types of medication.
*Average score: 3.25. Your previous rating: 2. Please confirm your selection.*
How much do you think clinical developments affect medication adherence?

○5 ○4 ○3 ○2 ○1

**13. Treatment Effectiveness**
Includes drug side effects, therapeutic effects, delayed response, and dosage accuracy.
*Average score: 3.5. Your previous rating: 3. Please confirm your selection.*
How much do you think treatment effectiveness affects medication adherence?

○5 ○4 ○3 ○2 ○1

**14. Doctor–Patient Communication**
Refers to communication between caregivers/family and healthcare providers.
*Average score: 3.75. Your previous rating: 3. Please confirm your selection.*
How much do you think doctor–patient communication affects medication adherence?

○5 ○4 ○3 ○2 ○1

**15. Economic Factors**
Includes treatment costs and the financial burden on the patient or family.
*Average score: 3.5. Your previous rating: 4. Please confirm your selection.*
How much do you think economic factors affect medication adherence?

○5 ○4 ○3 ○2 ○1

**16. Assistive Tools**
Refers to smart tools such as pill box dividers, medication organizers, or smart packaging.
*Average score: 3.25. Your previous rating: 4. Please confirm your selection.*
How much do you think assistive tools affect medication adherence?

○5 ○4 ○3 ○2 ○1

**17. Technology and Innovation**
Includes effective technologies such as artificial intelligence (AI), augmented reality (AR), and image processing.
*Average score: 3.25. Your previous rating: 3. Please confirm your selection.*
How much do you think technology and innovation affect medication adherence?

○5 ○4 ○3 ○2 ○1

**18. User Needs and Experience**
Refers to accessible design, clear information delivery, ease of understanding, and usability.
*Average score: 3.5. Your previous rating: 2. Please confirm your selection.*
How much do you think user needs and experience affect medication adherence?

○5 ○4 ○3 ○2 ○1

**19. Interactive Features and Formats**
Includes clear labeling and packaging, support platforms, healthcare accessibility and flexibility, and medication management apps (e.g., for supervision or information filtering).
*Average score: 3.63. Your previous rating: 3. Please confirm your selection.*
How much do you think interactive features and formats affect medication adherence?

○5 ○4 ○3 ○2 ○1

**20. Cultural Ideologies**
Includes beliefs about medication management, communication styles, acceptance of new products, and cultural values such as filial piety and hands-on caregiving.
*Average score: 3.75. Your previous rating: 2. Please confirm your selection.*
How much do you think cultural ideologies affect medication adherence?

○5 ○4 ○3 ○2 ○1

N7:

**Survey on Factors Influencing Medication Adherence in Patients with Alzheimer’s Disease**

Please review the explanation of each factor. The average score from the previous round and your previous response are shown. Kindly confirm your choice again.

**[Single-choice question: 5 = Very strong influence, 1 = Very little influence]**

**1. Cognitive Decline**
Mainly manifested as memory loss, impaired recall, and reduced executive function.
*Average score from the previous round: 3.88. Your previous rating: 5. Please confirm your selection.*
To what extent do you think cognitive decline affects medication adherence in Alzheimer’s patients?

○5 ○4 ○3 ○2 ○1

**2. Awareness of Treatment**
Without awareness, patients may resist medication in early stages and lack understanding of the need for treatment.
*Average score: 3.88. Your previous rating: 4. Please confirm your selection.*
To what extent do you think awareness of treatment affects medication adherence?

○5 ○4 ○3 ○2 ○1

**3. Treatment Motivation**
Includes psychological health, treatment beliefs and attitudes, and level of active participation.
*Average score: 3.75. Your previous rating: 4. Please confirm your selection.*
To what extent do you think treatment motivation affects medication adherence?

○5 ○4 ○3 ○2 ○1

**4. Complexity of the Treatment Plan**
Includes polypharmacy, complicated dosing schedules, challenges in dosage tracking, and non-oral administration.
*Average score: 3.25. Your previous rating: 4. Please confirm your selection.*
To what extent do you think treatment complexity affects medication adherence?

○5 ○4 ○3 ○2 ○1

**5. Patient Habits and Behaviors**
Includes harmful habits such as smoking and alcohol consumption.
*Average score: 4.25. Your previous rating: 5. Please confirm your selection.*
To what extent do you think personal habits and behaviors affect medication adherence?

○5 ○4 ○3 ○2 ○1

**6. Family and Social Support**
Includes support from family and the community, such as home-based care.
*Average score: 4.75. Your previous rating: 5. Please confirm your selection.*
To what extent do you think family and social support affect medication adherence?

○5 ○4 ○3 ○2 ○1

**7. Emotional Support**
Includes companionship, attitude toward treatment, and emotional understanding (e.g., encouragement).
*Average score: 4.25. Your previous rating: 4. Please confirm your selection.*
To what extent do you think emotional support affects medication adherence?

○5 ○4 ○3 ○2 ○1

**8. Treatment Assistance**
Includes medication supervision by family, help with medication management, and doctor–patient communication.
*Average score: 4.38. Your previous rating: 4. Please confirm your selection.*
To what extent do you think treatment assistance affects medication adherence?

○5 ○4 ○3 ○2 ○1

**9. Caregiver’s Professional Education**
Includes caregiving skills, effective communication, stress management, and understanding of Alzheimer’s and related medications.
*Average score: 4.38. Your previous rating: 3. Please confirm your selection.*
To what extent do you think caregiver education affects medication adherence?

○5 ○4 ○3 ○2 ○1

**10. Family Environment**
Includes family stability, level of cooperation in treatment, whether the patient lives alone, and general living conditions.
*Average score: 4.63. Your previous rating: 4. Please confirm your selection.*
To what extent do you think family environment affects medication adherence?

○5 ○4 ○3 ○2 ○1

**11. Disease Progression**
Includes adherence changes at different disease stages, cognitive decline, condition deterioration, treatment modifications, and treatment duration.
*Average score: 4.00. Your previous rating: 5. Please confirm your selection.*
To what extent do you think disease progression affects medication adherence?

○5 ○4 ○3 ○2 ○1

**12. Clinical Developments**
Refers to treatment strategies such as combination therapy and drug type.
*Average score: 3.25. Your previous rating: 5. Please confirm your selection.*
To what extent do you think clinical developments affect medication adherence?

○5 ○4 ○3 ○2 ○1

**13. Treatment Effectiveness**
Includes side effects, drug efficacy, delayed response, and dosage precision.
*Average score: 3.5. Your previous rating: 5. Please confirm your selection.*
To what extent do you think treatment effectiveness affects medication adherence?

○5 ○4 ○3 ○2 ○1

**14. Doctor–Patient Communication**
Refers to communication between caregivers/family and healthcare professionals.
*Average score: 3.75. Your previous rating: 5. Please confirm your selection.*
To what extent do you think doctor–patient communication affects medication adherence?

○5 ○4 ○3 ○2 ○1

**15. Economic Factors**
Includes treatment costs and financial burden.
*Average score: 3.5. Your previous rating: 5. Please confirm your selection.*
To what extent do you think economic factors affect medication adherence?

○5 ○4 ○3 ○2 ○1

**16. Assistive Tools**
Includes smart applications such as pill organizers, dividers, and intelligent packaging.
*Average score: 3.25. Your previous rating: 3. Please confirm your selection.*
To what extent do you think assistive tools affect medication adherence?

○5 ○4 ○3 ○2 ○1

**17. Technology and Innovation**
Includes advanced technologies like artificial intelligence, augmented reality, and image processing.
*Average score: 3.25. Your previous rating: 3. Please confirm your selection.*
To what extent do you think technology and innovation affect medication adherence?

○5 ○4 ○3 ○2 ○1

**18. User Needs and Experience**
Refers to accessibility, clarity of information, ease of use, and comprehensibility.
*Average score: 3.5. Your previous rating: 4. Please confirm your selection.*
To what extent do you think user needs and experience affect medication adherence?

○5 ○4 ○3 ○2 ○1

**19. Interactive Features and Formats**
Includes clear packaging and labeling, social support platforms, flexible healthcare access, and medication management apps (e.g., for monitoring or filtering information).
*Average score: 3.63. Your previous rating: 4. Please confirm your selection.*
To what extent do you think interactive features and formats affect medication adherence?

○5 ○4 ○3 ○2 ○1

**20. Cultural Ideologies**
Includes beliefs regarding medication management, communication styles, acceptance of new technologies, and cultural values (e.g., filial piety and hands-on caregiving).
*Average score: 3.75. Your previous rating: 4. Please confirm your selection.*
To what extent do you think cultural ideologies affect medication adherence?

○5 ○4 ○3 ○2 ○1

N8：

**Survey on Factors Influencing Medication Adherence in Patients with Alzheimer’s Disease**

Please review each factor description. The average score from the previous round and your previous rating are shown. Kindly confirm your response again.

**[Single-choice question: 5 = Very strong influence, 1 = Very little influence]**

**1. Cognitive Decline**
Mainly manifested as memory loss, cognitive impairment, and reduced executive functioning.
*Average score in previous round: 3.88. Your previous rating: 4. Please confirm your selection.*
To what extent do you think cognitive decline affects medication adherence?

○5 ○4 ○3 ○2 ○1

**2. Awareness of Treatment**
Patients without treatment awareness may initially resist medication and lack understanding of its necessity.
*Average score: 3.88. Your previous rating: 4. Please confirm your selection.*
To what extent do you think awareness of treatment affects medication adherence?

○5 ○4 ○3 ○2 ○1

**3. Treatment Motivation**
Includes psychological wellbeing, beliefs and attitudes toward treatment, and level of engagement.
*Average score: 3.75. Your previous rating: 4. Please confirm your selection.*
To what extent do you think treatment motivation affects medication adherence?

○5 ○4 ○3 ○2 ○1

**4. Complexity of the Treatment Plan**
Includes polypharmacy, complex dosing schedules, difficulty tracking dosage, and non-oral routes.
*Average score: 3.25. Your previous rating: 4. Please confirm your selection.*
To what extent do you think treatment complexity affects medication adherence?

○5 ○4 ○3 ○2 ○1

**5. Patient Habits and Behaviors**
Refers to poor habits such as smoking, alcohol consumption, etc.
*Average score: 4.25. Your previous rating: 5. Please confirm your selection.*
To what extent do you think personal habits and behaviors affect medication adherence?

○5 ○4 ○3 ○2 ○1

**6. Family and Social Support**
Includes support from family or community members, such as home care.
*Average score: 4.75. Your previous rating: 5. Please confirm your selection.*
To what extent do you think family and social support affect medication adherence?

○5 ○4 ○3 ○2 ○1

**7. Emotional Support**
Includes family presence, supportive attitudes, emotional understanding, and encouragement.
*Average score: 4.25. Your previous rating: 5. Please confirm your selection.*
To what extent do you think emotional support affects medication adherence?

○5 ○4 ○3 ○2 ○1

**8. Treatment Assistance**
Includes medication supervision, help with administration, and communication with healthcare professionals.
*Average score: 4.38. Your previous rating: 4. Please confirm your selection.*
To what extent do you think treatment assistance affects medication adherence?

○5 ○4 ○3 ○2 ○1

**9. Caregiver’s Professional Education**
Includes caregiving skills, effective communication, emotional support, and knowledge of the disease and medications.
*Average score: 4.38. Your previous rating: 3. Please confirm your selection.*
To what extent do you think caregiver education affects medication adherence?

○5 ○4 ○3 ○2 ○1

**10. Family Environment**
Includes family stability, involvement in care, whether the patient lives alone, and overall living conditions.
*Average score: 4.63. Your previous rating: 3. Please confirm your selection.*
To what extent do you think family environment affects medication adherence?

○5 ○4 ○3 ○2 ○1

**11. Disease Progression**
Includes adherence variations across stages, worsening cognitive symptoms, changes in regimen, and treatment duration.
*Average score: 4.00. Your previous rating: 4. Please confirm your selection.*
To what extent do you think disease progression affects medication adherence?

○5 ○4 ○3 ○2 ○1

**12. Clinical Developments**
Includes overall treatment strategies, types of medications used, and integration of therapies.
*Average score: 3.25. Your previous rating: 4. Please confirm your selection.*
To what extent do you think clinical developments affect medication adherence?

○5 ○4 ○3 ○2 ○1

**13. Treatment Effectiveness**
Refers to drug side effects, pharmacodynamics, delayed responses, and dosing accuracy.
*Average score: 3.5. Your previous rating: 4. Please confirm your selection.*
To what extent do you think treatment effectiveness affects medication adherence?

○5 ○4 ○3 ○2 ○1

**14. Doctor–Patient Communication**
Refers to interactions and information exchange between family members and healthcare professionals.
*Average score: 3.75. Your previous rating: 5. Please confirm your selection.*
To what extent do you think doctor–patient communication affects medication adherence?

○5 ○4 ○3 ○2 ○1

**15. Economic Factors**
Includes medication costs and the financial burden of treatment.
*Average score: 3.5. Your previous rating: 5. Please confirm your selection.*
To what extent do you think economic factors affect medication adherence?

○5 ○4 ○3 ○2 ○1

**16. Assistive Tools**
Refers to smart tools such as pill dividers, smart packaging, and medication organizers.
*Average score: 3.25. Your previous rating: 3. Please confirm your selection.*
To what extent do you think assistive tools affect medication adherence?

○5 ○4 ○3 ○2 ○1

**17. Technology and Innovation**
Includes helpful technologies such as artificial intelligence (AI), augmented reality (AR), and image processing.
*Average score: 3.25. Your previous rating: 2. Please confirm your selection.*
To what extent do you think technology and innovation affect medication adherence?

○5 ○4 ○3 ○2 ○1

**18. User Needs and Experience**
Includes accessibility of design, clarity of information, ease of understanding, and usability.
*Average score: 3.5. Your previous rating: 4. Please confirm your selection.*
To what extent do you think user needs and experience affect medication adherence?

○5 ○4 ○3 ○2 ○1

**19. Interactive Features and Format**
Includes packaging clarity, support platforms, healthcare flexibility, and medication management apps (e.g., reminders, data filtering).
*Average score: 3.63. Your previous rating: 3. Please confirm your selection.*
To what extent do you think interactive functions and formats affect medication adherence?

○5 ○4 ○3 ○2 ○1

**20. Cultural Ideologies**
Includes attitudes toward medication, communication styles, openness to new tools, and cultural values (e.g., filial piety and hands-on caregiving).
*Average score: 3.75. Your previous rating: 4. Please confirm your selection.*
To what extent do you think cultural ideologies affect medication adherence?

○5 ○4 ○3 ○2 ○1

N9：

**Survey on Factors Influencing Medication Adherence in Patients with Alzheimer’s Disease**

Please review the explanation of each factor. The average score from the previous round and your last response are provided. Kindly confirm your selection again.

**[Single-choice question: 5 = Very strong influence, 1 = Very little influence]**

**1. Cognitive Decline**
Primarily manifested as memory loss, cognitive impairment, and reduced executive function.
*Previous round average: 3.88. Your previous response: 5. Please confirm your choice.*
To what extent do you think cognitive decline affects medication adherence?

○5 ○4 ○3 ○2 ○1

**2. Awareness of Treatment**
Without awareness, patients may resist treatment in early stages and lack understanding of medication necessity.
*Average: 3.88. Your response: 5.*
To what extent does treatment awareness influence medication adherence?

○5 ○4 ○3 ○2 ○1

**3. Treatment Motivation**
Includes mental health, beliefs and attitudes toward treatment, and level of engagement.
*Average: 3.75. Your response: 4.*
To what extent does treatment motivation affect medication adherence?

○5 ○4 ○3 ○2 ○1

**4. Complexity of Treatment Plan**
Involves polypharmacy, frequent dosing, difficulty tracking doses, and non-oral administration routes.
*Average: 3.25. Your response: 4.*
To what extent does treatment complexity influence medication adherence?

○5 ○4 ○3 ○2 ○1

**5. Patient Habits and Behaviors**
Includes harmful habits such as smoking and alcohol consumption.
*Average: 4.25. Your response: 5.*
To what extent do habits and behaviors affect adherence?

○5 ○4 ○3 ○2 ○1

**6. Family and Social Support**
Includes care from family and community, such as home care services.
*Average: 4.75. Your response: 5.*
To what extent does family and social support affect adherence?

○5 ○4 ○3 ○2 ○1

**7. Emotional Support**
Includes companionship, emotional understanding, and encouragement during treatment.
*Average: 4.25. Your response: 5.*
To what extent does emotional support influence adherence?

○5 ○4 ○3 ○2 ○1

**8. Treatment Assistance**
Includes family supervision of medication, management help, and communication with healthcare professionals.
*Average: 4.38. Your response: 4.*
To what extent does treatment assistance affect adherence?

○5 ○4 ○3 ○2 ○1

**9. Caregiver’s Professional Education**
Refers to caregiving skills, communication, stress reduction, and understanding of the disease and medications.
*Average: 4.38. Your response: 4.*
To what extent does caregiver education influence adherence?

○5 ○4 ○3 ○2 ○1

**10. Family Environment**
Includes family stability, willingness to participate in care, living conditions, and whether the patient lives alone.
*Average: 4.63. Your response: 4.*
To what extent does the family environment affect adherence?

○5 ○4 ○3 ○2 ○1

**11. Disease Progression**
Includes stage-specific adherence, worsening condition, regimen changes, and treatment duration.
*Average: 4.00. Your response: 4.*
To what extent does disease progression affect adherence?

○5 ○4 ○3 ○2 ○1

**12. Clinical Developments**
Refers to new treatment strategies, combined medications, or drug categories.
*Average: 3.25. Your response: 4.*
To what extent do clinical developments affect adherence?

○5 ○4 ○3 ○2 ○1

**13. Treatment Effectiveness**
Includes side effects, drug response, delayed efficacy, and precise dosing.
*Average: 3.5. Your response: 5.*
To what extent does treatment effectiveness influence adherence?

○5 ○4 ○3 ○2 ○1

**14. Doctor–Patient Communication**
Refers to interactions between family caregivers and healthcare professionals.
*Average: 3.75. Your response: 5.*
To what extent does communication affect adherence?

○5 ○4 ○3 ○2 ○1

**15. Economic Factors**
Includes treatment costs and the financial burden on families.
*Average: 3.5. Your response: 5.*
To what extent do economic factors influence adherence?

○5 ○4 ○3 ○2 ○1

**16. Assistive Tools**
Refers to smart aids like pill organizers, packaging with reminders, etc.
*Average: 3.25. Your response: 4.*
To what extent do assistive tools affect adherence?

○5 ○4 ○3 ○2 ○1

**17. Technology and Innovation**
Refers to technologies such as AI, AR, image processing, etc.
*Average: 3.25. Your response: 3.*
To what extent do technologies and innovations influence adherence?

○5 ○4 ○3 ○2 ○1

**18. User Needs and Experience**
Includes accessible design, clear communication, and ease of use.
*Average: 3.5. Your response: 3.*
To what extent do user needs and experience affect adherence?

○5 ○4 ○3 ○2 ○1

**19. Interactive Functions and Formats**
Includes packaging clarity, support platforms, flexible care access, and medication apps (e.g., monitoring, filtering).
*Average: 3.63. Your response: 4.*
To what extent do interactive features affect adherence?

○5 ○4 ○3 ○2 ○1

**20. Cultural Ideologies**
Includes beliefs about medication, communication styles, openness to new products, and traditional caregiving values (e.g., filial piety).
*Average: 3.75. Your response: 5.*
To what extent does cultural ideology influence adherence?

○5 ○4 ○3 ○2 ○1

N10：

**Survey on Factors Influencing Medication Adherence in Patients with Alzheimer’s Disease**

Please confirm your selection again. For each item, the average score from the previous round and your previous selection are shown.
[Single-choice question: 5 = Very strong influence, 1 = Very little influence]

**1. Cognitive Decline**
Mainly characterized by memory loss, impaired memory function, and reduced executive ability.
*Previous round average: 3.88. Your previous choice: 4. Please confirm your choice.*
To what extent do you think cognitive decline affects medication adherence in Alzheimer’s patients?
○5 ○4 ○3 ○2 ○1

**2. Awareness of Treatment**
Patients may initially resist medication due to lack of awareness about treatment necessity.
*Average: 3.88. Your choice: 3.*
To what extent does treatment awareness affect medication adherence?
○5 ○4 ○3 ○2 ○1

**3. Treatment Motivation**
Includes psychological health, belief in treatment, attitude, and level of engagement.
*Average: 3.75. Your choice: 3.*
To what extent does motivation influence adherence?
○5 ○4 ○3 ○2 ○1

**4. Complexity of Treatment Regimen**
Refers to multiple medications, frequent dosing, difficulty tracking, or non-oral administration.
*Average: 3.25. Your choice: 4.*
To what extent does treatment complexity affect adherence?
○5 ○4 ○3 ○2 ○1

**5. Patient Habits and Behaviors**
Includes harmful habits such as smoking and drinking.
*Average: 4.25. Your choice: 4.*
To what extent do personal habits affect adherence?
○5 ○4 ○3 ○2 ○1

**6. Family and Social Support**
Includes care from family and community, such as home care.
*Average: 4.75. Your choice: 5.*
To what extent does social support affect adherence?
○5 ○4 ○3 ○2 ○1

**7. Emotional Support**
Includes companionship, supportive attitudes, and emotional understanding (e.g., encouragement).
*Average: 4.25. Your choice: 5.*
To what extent does emotional support influence adherence?
○5 ○4 ○3 ○2 ○1

**8. Assistance with Treatment**
Includes supervision by family, help with medication management, and communication with professionals.
*Average: 4.38. Your choice: 4.*
To what extent does assistance affect adherence?
○5 ○4 ○3 ○2 ○1

**9. Caregiver’s Professional Education**
Includes caregiving skills, communication, stress management, and knowledge of the disease and medication.
*Average: 4.38. Your choice: 4.*
To what extent does caregiver education influence adherence?
○5 ○4 ○3 ○2 ○1

**10. Family Environment**
Includes family stability, willingness to assist, living alone, and daily living conditions.
*Average: 4.63. Your choice: 5.*
To what extent does the family environment affect adherence?
○5 ○4 ○3 ○2 ○1

**11. Disease Progression**
Includes stage-specific adherence, cognitive decline, worsening condition, and treatment changes.
*Average: 4.00. Your choice: 4.*
To what extent does disease progression affect adherence?
○5 ○4 ○3 ○2 ○1

**12. Clinical Advancements**
Refers to new treatment strategies or changes in medication types.
*Average: 3.25. Your choice: 4.*
To what extent do clinical changes affect adherence?
○5 ○4 ○3 ○2 ○1

**13. Treatment Effectiveness**
Includes drug side effects, efficacy, delayed onset, and dosage precision.
*Average: 3.5. Your choice: 5.*
To what extent does treatment effectiveness affect adherence?
○5 ○4 ○3 ○2 ○1

**14. Doctor–Patient Communication**
Refers to communication between doctors and family caregivers.
*Average: 3.75. Your choice: 5.*
To what extent does communication affect adherence?
○5 ○4 ○3 ○2 ○1

**15. Economic Factors**
Includes treatment costs and financial burden.
*Average: 3.5. Your choice: 5.*
To what extent do economic concerns affect adherence?
○5 ○4 ○3 ○2 ○1

**16. Assistive Tools**
Such as smart pill boxes, divided organizers, and intelligent packaging.
*Average: 3.25. Your choice: 4.*
To what extent do assistive tools influence adherence?
○5 ○4 ○3 ○2 ○1

**17. Technology and Innovation**
Such as AI, augmented reality, or image recognition technologies.
*Average: 3.25. Your choice: 3.*
To what extent do innovations affect adherence?
○5 ○4 ○3 ○2 ○1

**18. User Needs and Experience**
Refers to accessibility, clarity of information, and ease of operation.
*Average: 3.5. Your choice: 5.*
To what extent does user experience influence adherence?
○5 ○4 ○3 ○2 ○1

**19. Interactive Functions and Features**
Includes clear packaging and labeling, support platforms, flexible services, and medication apps.
*Average: 3.63. Your choice: 4.*
To what extent do interactive functions affect adherence?
○5 ○4 ○3 ○2 ○1

**20. Cultural Ideologies**
Includes attitudes toward medication, communication styles, openness to new tools, and family traditions (e.g., filial piety).
*Average: 3.75. Your choice: 4.*
To what extent does cultural ideology influence adherence?
○5 ○4 ○3 ○2 ○1

N11：

**Survey on Factors Influencing Medication Adherence in Patients with Alzheimer’s Disease**

Please confirm your selection again. For each item, the average score from the previous round and your previous selection are shown.
[Single-choice question: 5 = Very strong influence, 1 = Very little influence]

**1. Cognitive Decline**
Mainly characterized by memory loss, impaired memory function, and reduced executive ability.
*Previous round average: 3.88. Your previous choice: 4. Please confirm your choice.*
To what extent do you think cognitive decline affects medication adherence in Alzheimer’s patients?
○5 ○4 ○3 ○2 ○1

**2. Awareness of Treatment**
Patients may initially resist medication due to lack of awareness about treatment necessity.
*Average: 3.88. Your choice: 3.*
To what extent does treatment awareness affect medication adherence?
○5 ○4 ○3 ○2 ○1

**3. Treatment Motivation**
Includes psychological health, belief in treatment, attitude, and level of engagement.
*Average: 3.75. Your choice: 3.*
To what extent does motivation influence adherence?
○5 ○4 ○3 ○2 ○1

**4. Complexity of Treatment Regimen**
Refers to multiple medications, frequent dosing, difficulty tracking, or non-oral administration.
*Average: 3.25. Your choice: 4.*
To what extent does treatment complexity affect adherence?
○5 ○4 ○3 ○2 ○1

**5. Patient Habits and Behaviors**
Includes harmful habits such as smoking and drinking.
*Average: 4.25. Your choice: 4.*
To what extent do personal habits affect adherence?
○5 ○4 ○3 ○2 ○1

**6. Family and Social Support**
Includes care from family and community, such as home care.
*Average: 4.75. Your choice: 5.*
To what extent does social support affect adherence?
○5 ○4 ○3 ○2 ○1

**7. Emotional Support**
Includes companionship, supportive attitudes, and emotional understanding (e.g., encouragement).
*Average: 4.25. Your choice: 5.*
To what extent does emotional support influence adherence?
○5 ○4 ○3 ○2 ○1

**8. Assistance with Treatment**
Includes supervision by family, help with medication management, and communication with professionals.
*Average: 4.38. Your choice: 4.*
To what extent does assistance affect adherence?
○5 ○4 ○3 ○2 ○1

**9. Caregiver’s Professional Education**
Includes caregiving skills, communication, stress management, and knowledge of the disease and medication.
*Average: 4.38. Your choice: 4.*
To what extent does caregiver education influence adherence?
○5 ○4 ○3 ○2 ○1

**10. Family Environment**
Includes family stability, willingness to assist, living alone, and daily living conditions.
*Average: 4.63. Your choice: 5.*
To what extent does the family environment affect adherence?
○5 ○4 ○3 ○2 ○1

**11. Disease Progression**
Includes stage-specific adherence, cognitive decline, worsening condition, and treatment changes.
*Average: 4.00. Your choice: 4.*
To what extent does disease progression affect adherence?
○5 ○4 ○3 ○2 ○1

**12. Clinical Advancements**
Refers to new treatment strategies or changes in medication types.
*Average: 3.25. Your choice: 4.*
To what extent do clinical changes affect adherence?
○5 ○4 ○3 ○2 ○1

**13. Treatment Effectiveness**
Includes drug side effects, efficacy, delayed onset, and dosage precision.
*Average: 3.5. Your choice: 5.*
To what extent does treatment effectiveness affect adherence?
○5 ○4 ○3 ○2 ○1

**14. Doctor–Patient Communication**
Refers to communication between doctors and family caregivers.
*Average: 3.75. Your choice: 5.*
To what extent does communication affect adherence?
○5 ○4 ○3 ○2 ○1

**15. Economic Factors**
Includes treatment costs and financial burden.
*Average: 3.5. Your choice: 5.*
To what extent do economic concerns affect adherence?
○5 ○4 ○3 ○2 ○1

**16. Assistive Tools**
Such as smart pill boxes, divided organizers, and intelligent packaging.
*Average: 3.25. Your choice: 4.*
To what extent do assistive tools influence adherence?
○5 ○4 ○3 ○2 ○1

**17. Technology and Innovation**
Such as AI, augmented reality, or image recognition technologies.
*Average: 3.25. Your choice: 3.*
To what extent do innovations affect adherence?
○5 ○4 ○3 ○2 ○1

**18. User Needs and Experience**
Refers to accessibility, clarity of information, and ease of operation.
*Average: 3.5. Your choice: 5.*
To what extent does user experience influence adherence?
○5 ○4 ○3 ○2 ○1

**19. Interactive Functions and Features**
Includes clear packaging and labeling, support platforms, flexible services, and medication apps.
*Average: 3.63. Your choice: 4.*
To what extent do interactive functions affect adherence?
○5 ○4 ○3 ○2 ○1

**20. Cultural Ideologies**
Includes attitudes toward medication, communication styles, openness to new tools, and family traditions (e.g., filial piety).
*Average: 3.75. Your choice: 4.*
To what extent does cultural ideology influence adherence?
○5 ○4 ○3 ○2 ○1

N12：

**Survey on Factors Influencing Medication Adherence in Patients with Alzheimer’s Disease**

For each of the following factors, the average score from the previous round is provided along with your previous selection.
Please confirm your choice again:
**[Single-choice question: 5 = Very strong influence, 1 = Very weak influence]**

**1. Cognitive Decline:**
Mainly manifests as memory deterioration, memory impairment, and decreased executive function.
*Previous round average: 3.88 | Your previous choice: 5*
**To what extent do you think cognitive decline affects medication adherence in Alzheimer’s patients?**
○5 ○4 ○3 ○2 ○1

**2. Awareness of Treatment:**
Without awareness of the need for treatment, patients may resist medication in early stages.
*Average: 3.88 | Your choice: 5*
**To what extent does awareness of treatment affect adherence?**
○5 ○4 ○3 ○2 ○1

**3. Motivation for Treatment:**
Includes psychological well-being, beliefs and attitudes toward treatment, and engagement in treatment.
*Average: 3.75 | Your choice: 4*
**To what extent does treatment motivation affect adherence?**
○5 ○4 ○3 ○2 ○1

**4. Complexity of Treatment Regimen:**
Multiple medications, complex dosing schedules, difficult unit tracking, or non-oral administration.
*Average: 3.25 | Your choice: 4*
**To what extent does treatment complexity affect adherence?**
○5 ○4 ○3 ○2 ○1

**5. Patient Habits and Behaviors:**
Includes harmful behaviors such as smoking and drinking.
*Average: 4.25 | Your choice: 5*
**To what extent do patient habits and behaviors affect adherence?**
○5 ○4 ○3 ○2 ○1

**6. Family and Social Support:**
Care provided by family or the community, such as home care.
*Average: 4.75 | Your choice: 5*
**To what extent does family and social support affect adherence?**
○5 ○4 ○3 ○2 ○1

**7. Emotional Support:**
Includes family companionship, attitudes toward treatment, emotional understanding, and encouragement.
*Average: 4.25 | Your choice: 5*
**To what extent does emotional support affect adherence?**
○5 ○4 ○3 ○2 ○1

**8. Treatment Assistance:**
Includes family monitoring of medication, helping manage medication, and communication with healthcare providers.
*Average: 4.38 | Your choice: 4*
**To what extent does assistance with treatment affect adherence?**
○5 ○4 ○3 ○2 ○1

**9. Caregiver’s Professional Education:**
Caregiving skills, effective communication, stress reduction, understanding of the disease and medication by the patient and their family, all of which may affect adherence.
*Average: 4.38 | Your choice: 4*
**To what extent does caregiver education affect adherence?**
○5 ○4 ○3 ○2 ○1

**10. Family Environment:**
Includes family stability, active participation of family members in treatment, whether the patient lives alone, and their living conditions.
*Average: 4.63 | Your choice: 4*
**To what extent does the family environment affect adherence?**
○5 ○4 ○3 ○2 ○1

**11. Disease Progression:**
Stage-specific adherence, cognitive decline, worsening condition, treatment adjustments, and long-term treatment duration.
*Average: 4.00 | Your choice: 4*
**To what extent does disease progression affect adherence?**
○5 ○4 ○3 ○2 ○1

**12. Clinical Advancements:**
Treatment strategies, combination therapies, and changes in drug types.
*Average: 3.25 | Your choice: 4*
**To what extent do clinical developments affect adherence?**
○5 ○4 ○3 ○2 ○1

**13. Treatment Effectiveness:**
Drug side effects, drug efficacy, delayed effects, and precise dosing.
*Average: 3.5 | Your choice: 5*
**To what extent does treatment effectiveness affect adherence?**
○5 ○4 ○3 ○2 ○1

**14. Doctor–Patient Communication:**
Communication between doctors and family caregivers.
*Average: 3.75 | Your choice: 5*
**To what extent does communication with healthcare providers affect adherence?**
○5 ○4 ○3 ○2 ○1

**15. Economic Factors:**
Treatment costs and financial burden.
*Average: 3.5 | Your choice: 5*
**To what extent do economic factors affect adherence?**
○5 ○4 ○3 ○2 ○1

**16. Assistive Tools:**
Smart tools such as pill box dividers, intelligent packaging, etc.
*Average: 3.25 | Your choice: 4*
**To what extent do assistive tools affect adherence?**
○5 ○4 ○3 ○2 ○1

**17. Technology and Innovation:**
Technologies such as AI, AR, image processing, etc.
*Average: 3.25 | Your choice: 3*
**To what extent do technology and innovation affect adherence?**
○5 ○4 ○3 ○2 ○1

**18. User Needs and Experience:**
Includes accessible design, clarity of information, and ease of operation.
*Average: 3.5 | Your choice: 3*
**To what extent do user needs and experience affect adherence?**
○5 ○4 ○3 ○2 ○1

**19. Interactive Features and Formats:**
Clear drug packaging and labeling, social support platforms, healthcare accessibility and flexibility, medication management apps (including supervision, information filtering, etc.).
*Average: 3.63 | Your choice: 4*
**To what extent do interactive features and formats affect adherence?**
○5 ○4 ○3 ○2 ○1

**20. Cultural Ideology:**
Beliefs around medication, communication preferences, openness to new products, cultural values (e.g., filial piety and hands-on caregiving).
*Average: 3.75 | Your choice: 5*
**To what extent does cultural ideology affect adherence?**
○5 ○4 ○3 ○2 ○1
