# Supplementary material for: Exploring Influencing Factors of Medication Adherence Among Chinese Patients With Alzheimer Disease: Delphi Study Informing Future Artificial Intelligence–Supported Interventions
Source: JMIR Form Res. 2026 Apr 17;10:e89508. doi: 10.2196/89508 (PMC13100466; doi:10.2196/89508)
Supplement: Checklist 1 [file formative-v10-e89508-s004.docx]

**Delphi studies in social and health sciences – recommendations for an interdisciplinary standardized reporting (DELPHISTAR).**

**This reporting guideline is meant for studies using Delphi techniques in the health and social sciences.** These also include all Delphi variants and modifications that meet the following criteria:

1. Survey of several people with specialized knowledge (e.g., operational knowledge, experiential knowledge, functional knowledge, contextual knowledge);
2. Structured communication process that involves a group of people with relevant expertise;
3. Carrying out at least two survey rounds or the option to respond at least two times;
4. Feedback: the (interim) results are presented to the respondents starting in the second round;
5. Basis is a quantitative questionnaire with the possibility to contribute or supplement arguments for the respective position;
6. All answers, quantitative and qualitative, are systematically analyzed (quantitative: e.g., descriptive statistics, qualitative: e.g., thematic analysis).

| **Topic** | **Section** | **Item** | **Checklist Item** | **Location where item is reported** | **Exemplary answer** |
| --- | --- | --- | --- | --- | --- |
| **I**  **Title and Abstract** |  | 1 | Identification as a Delphi procedure in the title | Exploring Influencing Factors of Medication Adherence among Chinese Alzheimer’s Disease Patients : A Delphi Study Informing Future AI-Supported Interventions | What is a public health intervention? Results of a Delphi study. |
|  |  | 2 | Identification as a Delphi procedure in the abstract | The exploratory online Delphi study with no modification predicted the key factors influencing medication adherence and based on the results confirmed the potential of AI to improve adherence. | A Delphi procedure was selected to answer the research question. |
|  |  | 3 | Structured abstract | You can find in abstract | e.g., background, method, results and discussion |
| **II**  **Context** | **Formal** | 4 | Information about the sources of funding | No funding support | The Delphi study was funded by [SOURCE]. |
|  |  | 5 | Information about the team of authors and/or researchers (e.g., discipline, institution) | The Delphi study was conducted by an interdisciplinary team with representatives from medicine, caregivers, and healthcare designer. | The Delphi study was conducted by an interdisciplinary team with representatives from medicine, public health, and health promotion. |
|  |  | 6 | Information about method consulting | No outside consulting about method took place. | The study group was advised by external experts from [INSTITUTION] regarding statistics.  Or:  No outside consulting in regard to method took place. |
|  |  | 7 | Information about the project background | The Delphi survey was conducted as part of a mixed-methods study aiming to assess and validate key factors affecting medication adherence among Chinese patients with Alzheimer’s disease, identify core needs related to patient-centred medication management, and explore potential opportunities for AI-supported optimisation of medication management. | The Delphi survey was part of a mixed-methods study on [AIM]. |
|  |  | 8 | Information about the study protocol | No formal study protocol was published for this Delphi study; however, the study design and procedures were predefined and are fully described in the Methods section. | The study protocol is available at [LINK]. |
|  | **Content** | 9 | Justification of the chosen method (Delphi procedure) to answer the research question | The Delphi method is suitable for answering the research question because it systematically gathers the judgments of different expert groups and can identity agreement and disagreement. | The Delphi method is suitable for answering the research question because it systematically gathers the judgments of different expert groups and can identity agreement and disagreement. |
|  |  | 10 | Aim of the Delphi procedure (e.g., consensus, forecasting) | The aim of the Delphi study was to reach expert consensus on the key factors affecting medication adherence among Chinese patients with Alzheimer’s disease | The aim of the Delphi study is to find consensus on criteria to define a public health intervention. |
| **III**  **Method** | **Body & Integration of knowledge** | 11 | Identification and elucidation of relevant expertise, spheres of experience, and perspectives (e.g., theory, practice, affected groups, disciplines) | Experts were selected to represent diverse perspectives relevant to medication adherence in Alzheimer’s disease, including family caregiving, clinical practice, organisational management, and design or research expertise. This multidisciplinary composition enabled the Delphi process to integrate practical caregiving experiences, clinical knowledge, service and organisational insights, and design-oriented perspectives on patient-centred medication management. | The experts should represent the sciences and clinical practice because [REASON]. |
|  |  | 12 | Handling of knowledge, expertise and perspectives which are missing or have been deliberately not integrated | Notably, although the participants did not have direct practical experience with AI, artificial intelligence was spontaneously raised during discussions with smart packaging experts as a potential support tool. This led to a more active and constructive exploration of its future applications in health management and packaging interaction. | If it is not possible to recruit experts specialized in [AREA], this is openly communicated to the other respondents during the Delphi process. |
|  |  | 13 | Basic definition of expert^1^ | An expert was defined as an individual with relevant practical or professional experience in Alzheimer’s disease care, medication management, or related research or design fields. | A person who has been active in the area for at least [NUMBER] years is considered to be an expert. |
|  | **Delphi variations** | 14 | Identification of the type of Delphi procedure and potential modifications (e.g., classic Delphi, real-time Delphi, group Delphi) | A classic Delphi procedure with no modifications was used, and the study was designed and reported in accordance with the DelphiSTAR guideline (Niederberger et al., 2024). | A classic Delphi procedure was used [LITERATURE REFERENCE]. |
|  |  | 15 | Justification of the Delphi variation and modifications, including during the Delphi process, if applicable | A classic Delphi procedure with no modifications was adopted in this study. This approach was considered appropriate because the research aimed to identify and prioritise key factors affecting medication adherence through structured, iterative rounds of expert feedback, which aligns with the traditional purpose of the Delphi method. | If the willingness to participate clearly decreases between the first and second rounds, a third round will not be held. |
|  | **Sample of experts** | 16 | Selection criteria for the experts (per round if there are different expert groups) | Specifically, participants were required to meet at least one of the following criteria: (1) having a professional background related to Alzheimer’s disease or healthcare practice (e.g., clinicians or healthcare professionals); (2) possessing substantial practical experience in Alzheimer’s disease care, such as family caregivers or individuals involved in long-term patient support; (3) holding roles related to health policy, organisational decision-making, or service management; or (4) having interdisciplinary experience relevant to health technology, such as the design or development of digital or smart healthcare solutions.  Participants with specific experience in applying AI to medication management were given priority. However, due to the emerging nature of the field, no suitable candidates with such expertise were identified. | All of the experts who met the definition were invited to the first round.  All of the experts who completed the previous round were invited to participate in the subsequent round. |
|  |  | 17 | Identification of the experts | Experts were identified through online recruitment via social media and professional networks related to Alzheimer’s disease care, healthcare practice, and design. Potential participants were screened based on predefined inclusion criteria to ensure their relevance to the research aims. | The experts were identified based on publications in [DATABASE]. |
|  |  | 18 | Information about recruiting and any subsequent recruiting of experts | Participants were recruited through online advertisements posted on social media and professional networks. A total of 12 experts were selected from an initial pool of candidates based on predefined inclusion criteria. The same panel of experts was invited to participate in all subsequent Delphi rounds, and no additional participants were recruited. | The experts were informed about the Delphi study and invited to participate. |
|  | **Survey** | 19 | Elucidation of the content development for the questionnaire^2^ | The questionnaire was developed based on a comprehensive literature review and the results of the first-round semi-structured interviews. Thematic analysis was conducted to identify core factors influencing medication adherence, and these factors were subsequently translated into structured questionnaire items for the following Delphi rounds. | The questionnaire was developed based on the results of systematic reviews [LITERATURE REFERENCE]. |
|  |  | 20 | Description of the questionnaire (content and structure) | The Delphi questionnaire consisted of two stages. In the first round, semi-structured interview questions were used to explore participants’ experiences and perspectives on medication adherence, family roles, patient behaviours, and medication management.  Based on the thematic analysis of the first-round data, 20 key influencing factors were identified. In the second and third rounds, these factors were transformed into structured questionnaire items and evaluated using a 5-point Likert scale to assess their perceived importance. In the final round, participants were provided with feedback from the previous round and invited to confirm or adjust their ratings. | The questionnaire was divided into three segments on [TOPICS]. The statements made in the questionnaire were evaluated using standardized items, with the option to comment in free-text boxes. |
|  | **Delphi rounds** | 21 | Number of Delphi rounds | 3 | Three Delphi rounds were held. |
|  |  | 22 | Information about the aims of the individual Delphi rounds | The first Delphi round aimed to explore key factors influencing medication adherence through semi-structured interviews and to develop an initial thematic framework.  The second round aimed to evaluate and prioritise the identified factors using a structured Likert-scale questionnaire.  The third round aimed to provide feedback from the previous round and allow participants to confirm or adjust their ratings, thereby supporting the formation and stabilisation of expert consensus. | The first Delphi round focused on exploring relevant aspects. These aspects were then presented to the experts in the second Delphi round for standardized evaluation. |
|  |  | 23 | Disclosure and justification of the criterion for discontinuation | The Delphi process was designed to include three rounds. The procedure was discontinued after the third round, as the predefined number of rounds had been completed and the results between the second and third rounds showed no statistically significant differences, indicating stability in expert ratings. | The number of rounds was defined in advance to be a maximum of three rounds. |
|  | **Feedback** | 24 | Information about what data was reported back per round | In the subsequent Delphi round, participants were provided with the mean scores of each factor from the previous round. This feedback allowed them to review the group responses and confirm or adjust their ratings. | In terms of feedback, we shared the statistical results plus the summary of the open responses. |
|  |  | 25 | Information on how the results of the previous Delphi round were fed back to the experts surveyed (e.g., via frequencies, mean values, measures of dispersion, listing of comments) | The results of the previous Delphi round were fed back to participants in the form of mean scores for each factor. | Mean values, standard deviations and percentage frequency distributions were reported. |
|  |  | 26 | Information on whether feedback was differentiated by specific groups (e.g., by field of expertise, institutional affiliation) | Feedback was aggregated across all experts. The results were not differentiated by specific groups or fields of expertise. | The feedback was aggregated across all expert groups. |
|  |  | 27 | Information about how dissent and unclear results were handled | In cases of dissent or unclear results, the factors were presented again in the subsequent Delphi round with feedback from the previous round. Participants were invited to review the group responses and adjust their ratings. Stability between rounds was assessed using the Wilcoxon signed-rank test. | The results showing dissent were presented again for evaluation in the next Delphi round. |
|  | **Data analysis** | 28 | Disclosure of the quantitative and qualitative analytical strategy | Qualitative data from the first Delphi round were analysed using thematic analysis, with manual coding to identify key factors influencing medication adherence. Inter-rater reliability was assessed using Cohen’s Kappa.  Quantitative data from the second and third rounds were analysed using descriptive statistics, including mean scores and ranking of factors. The Wilcoxon signed-rank test was used to assess the stability of responses between rounds. | The quantitative items were descriptively analyzed. The open-ended items were analyzed using thematic analysis [LITERATURE REFERENCE]. |
|  |  | 29 | Definition and measurement of consensus | Consensus was defined in terms of stability between Delphi rounds. The Wilcoxon signed-rank test was used to compare results between rounds two and three. A non-significant difference indicated stability in expert ratings and was interpreted as evidence of consensus. | Consensus was defined as percentage agreement, meaning that agreement was assumed if at least 80% of the respondents agreed on an item. |
|  |  | 30 | Information on group-specific analysis or weighting of experts (e.g., theory vs. practice, discipline-specific analysis) | No group-specific analysis or weighting of experts was applied. All expert responses were treated equally in the analysis. | In the analysis, the mean values for percent agreement are weighted for each expert group in terms of the number of group members. |
| **IV**  **Results** | **Delphi process** | 31 | Illustration of the Delphi process (e.g., in a flow chart) | The Delphi process consisted of three sequential rounds. In the first round, semi-structured interviews were conducted to explore key factors influencing medication adherence among Chinese patients with Alzheimer’s disease. The interview data were analysed using thematic analysis, which resulted in the identification of 20 core influencing factors.  In the second round, these factors were converted into structured questionnaire items and evaluated using a 5-point Likert scale to assess their relative importance. Mean scores were calculated for each factor.  In the third round, participants were provided with feedback in the form of mean scores from the previous round and were invited to confirm or adjust their ratings. The stability of responses between rounds was assessed using the Wilcoxon signed-rank test to determine whether consensus had been reached. | A summary of the process is illustrated in a flow chart (Figure 1). |
|  |  | 32 | Information about special aspects during the Delphi process (e.g., deviations from the intended approach with justification) | No major deviations from the planned Delphi procedure were observed. The study was conducted as originally designed, with the same panel participating across all three rounds. | During the Delphi procedures the political discussion mentioned climate change and the effects on health. It is possible that this influenced the experts' responses. |
|  |  | 33 | Number of experts per round (both invited and participating) | A total of 12 experts were invited to participate in the Delphi study. All 12 experts completed round 1, and the same panel participated in rounds 2 and 3, resulting in a 100% response rate across all rounds. | The number of experts participating in the first Delphi round was [NUMBER], and the number of experts in the second round was [NUMBER]. This corresponds to a response rate of [NUMBER]% in the first round and [NUMBER]% in the second round. |
|  | **Results** | 34 | Presentation of the results for each Delphi round and the final results | The results of each Delphi round, including the qualitative findings from round 1, the quantitative ratings from round 2, and the final consensus results from round 3, are presented in the Results section. | In the first Delphi round [NUMBER]% of the respondents agreed, in the second [NUMBER]%, and in the third [NUMBER]%. |
| **V Discussion** | **Quality of findings** | 35 | Highlighting the findings from the Delphi study | The Delphi study identified a set of key factors influencing medication adherence among Chinese patients with Alzheimer’s disease, highlighting the central role of family support, medication complexity, disease progression, and emotional and cognitive conditions. These findings suggest that medication adherence is shaped by a complex interaction of clinical, caregiving, and behavioural factors rather than by a single determinant.  In addition, the results revealed two major opportunities for AI-supported interventions: precision therapy to address medication complexity and treatment variability, and AI-enabled educational support to assist caregivers in managing medication-related tasks. | The central findings can be summarized as follows: [STATE FINDINGS]. |
|  |  | 36 | Validity of the results (e.g., transferability of the findings) | The findings of this Delphi study are context-specific and should be interpreted within the Chinese caregiving and healthcare environment. As the expert panel was relatively small and purposively selected, the results should be considered exploratory and may not be directly transferable to other cultural or healthcare contexts without further validation. | The results are not transferable to other countries due to different legal regulations. |
|  |  | 37 | Reliability of the results (e.g., split half, inter-rater reliability) | Reliability was supported through both qualitative and quantitative procedures. In the first Delphi round, qualitative data were independently coded by two researchers, and inter-rater reliability was assessed using Cohen’s Kappa. In the quantitative rounds, the stability of expert ratings between rounds two and three was examined using the Wilcoxon signed-rank test. | The responses in the free-text comments were analyzed by two independent reviewers [SPECIFY]. |
|  |  | 38 | Reflection on potential limitations (e.g., distortion, skewing, bias) | Several limitations should be acknowledged. First, the expert panel was relatively small, which may limit the generalisability of the findings. Second, the panel composition was uneven, with a higher proportion of caregivers than clinical professionals, which may have influenced the prioritisation of certain factors. Third, the study was conducted within the Chinese healthcare and caregiving context, which may restrict the transferability of the results to other settings. Finally, although AI-related opportunities were identified, the panel did not include experts with direct practical experience in AI, and these findings should therefore be interpreted as exploratory. | The results are to be viewed critically with regard to the composition of the panel because [REASONS]. |

^1^ “Experts” are the participants; this can be people from academia, practice, or representatives of lived experience (e.g., patients, family members).

^2^ The term “questionnaire” stands for the survey instrument regardless of whether quantitative or qualitative items are integrated or weighted.

**Contact**

Prof. Dr. Marlen Niederberger

E-mail: marlen.niederberger(at)ph-gmuend.de

Department of Research Methods in Health Promotion and Prevention, Institute for Health Sciences

University of Education Schwäbisch Gmünd, Oberbettringer Straße 200, 73525 Schwäbisch Gmünd, Germany
